# Fully Selective Synthesis of Spirocyclic-1,2-oxazine N-Oxides via Non-Catalysed Hetero Diels-Alder Reactions with the Participation of Cyanofunctionalysed Conjugated Nitroalkenes

**DOI:** 10.3390/molecules28124586

**Published:** 2023-06-06

**Authors:** Przemysław Woliński, Agnieszka Kącka-Zych, Aneta Wróblewska, Ewelina Wielgus, Rafał Dolot, Radomir Jasiński

**Affiliations:** 1 Department of Organic Chemistry and Technology, Cracow University of Technology, Warszawska 24, 31-155 Krakow, Poland; przemyslaw.wolinski@pk.edu.pl; 2 Department of Organic Chemistry, University of Lodz, Tamka 12, 91-403 Lodz, Poland; aneta.wroblewska@chemia.uni.lodz.pl; 3 Centre of Molecular and Macromolecular Studies, Polish Academy of Sciences, Sienkiewicza 112, 90-363 Lodz, Poland; ms@cbmm.lodz.pl (E.W.); rdolot@cbmm.lodz.pl (R.D.)

**Keywords:** Hetero Diels-Alder reaction, nitroalkenes, heterocycles, molecular electron density theory, conceptual density functional theory

## Abstract

Hetero Diels-Alder (HDA) reactions with the participation of E-2-aryl-1-cyano-1-nitroethenes and methylenecyclopentane were evaluated on the basis of experimental as well as quantumchemical data. It was found that contrary to most known HDA reactions, title processes are realised under non-catalytic conditions and with full regiocontrol. The DFT study shows, without any doubt, the polar but single-step reaction mechanism. Deeper exploration using Bonding Evolution Theory (BET) techniques gives a clear image of the sequences of electron density reorganisation along the reaction coordinate. The first C4-C5 bond is created in phase *VII* by merging two monosynaptic basins, while the second O1-C6 bond is created in the last phase by a donation of the nonbonding electron density of O1 to C6. Based on the research, we can conclude that the analysed reaction proceeds according to a two-stage one-step mechanism.

## 1. Introduction

Six-membered heterocycles are a crucial and interesting class of compounds among the applied branches of organic chemistry, with a meaningful amount of research dedicated to the development of novel molecules [1,2,3]. Numerous organic synthesis protocols have been developed, which found applications in the chemical sciences. Many heterocyclic compounds occur naturally, for example, nucleic acids [4,5] or vitamins [6,7,8,9]. Six-membered heterocycles are valuable molecular systems from a practical point of view. These compounds are mainly used in medicinal chemistry [10,11,12] and agrochemical products [13,14]. Applications are also found in developers [6] as corrosion inhibitors [15,16], and this type of heterocyclic skeleton exists inter’alia within alkaloid pigments such as trichotomine [17].

1,2-Oxazine molecular systems are found to be potential precursors for the synthesis of biologically active compounds due to the highly reactive N-O bond [18]. Compounds including this-type heterocyclic skeleton have found application as thermochromic agents [19], anticancer [20], and antibacterials [21]. It is interesting that based on the herbicidal testing, oxazine derivatives possessed excellent herbicidal activity agents at the root of radish, grain sorghum, rape, cucumber and barnyard grass [22]. This class of compounds show high herbicidal activity on a wide variety of grasses and significant safety in cereals, rice, soybeans, and corn in preemergence tests [23]. Generally, the universal strategy for constructing six-membered organic rings is the Diels-Alder (DA) reaction [24]. With the participation of the buta-1,3-diene, cyclopentadiene, cyclohexadiene, and other conjugated dienes, these-type reactions lead very easily to respective analogues of cyclohexene or cyclohexadiene. This is especially easy in the case of the addition of mentioned dienes to electrophilically activated alkenes or alkynes [25]:


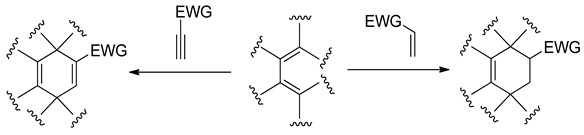


Based on hetero-analogues of conjugated dienes, preparing of six-membered heterocycles is possible via Hetero Diels-Alder (HDA) reaction. One of the most important hetero-analogues of dienes in organic synthesis is nitroethene and its analogues [26]. Many examples of these transformations were described in the second part of the XX century [27,28,29]. It is, however, generally known that for the contrast of the “carbo” DA reactions, HDA processes involving conjugated nitroalkenes as analogues of dienes require the participation of Lewis acid catalysts. For example, the HDA reaction between nitrocyclohexene and cyclopentene requires the presence of tin tetrachloride [30]:


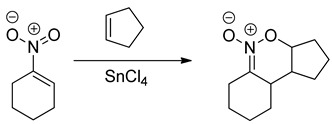


In the framework of this work, we described a rare case of non-catalysed HDA reaction with the participation of E-2-phenyl-1-cyano-1-nitroethene (**1c**) and its substituted analogues (**1a**,**b**,**d**–**g**). This group of nitroalkenes was discovered in the first half of the XX century [31], but its physicochemistry has yet to be well known. In particular, some information regarding the participation of E-2-aryl-1-cyano-1-nitroethenes in [3 + 2] cycloaddition processes [32,33], as well as DA reaction with cyclopentadiene [34] are available. In particular, we decided to try the synthesis of new spirocyclic HDA adducts, which were not yet prepared in this way. For this purpose, as a 2π-component, we selected methylenecyclopentane (**2**). The experimental studies were supplemented with the data obtained from Density Functional Theory (DFT) computational study.

## 2. Results and Discussion

**Scheme 1:**
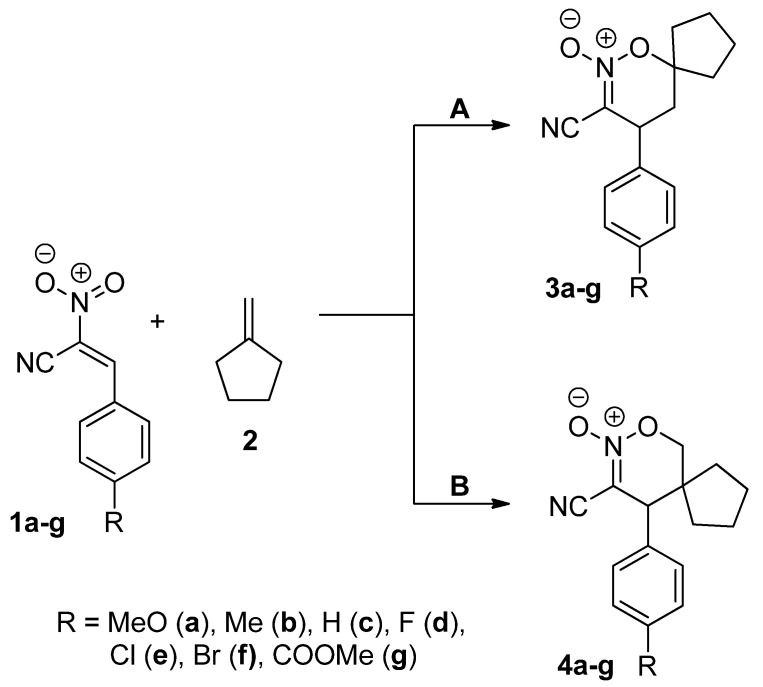
HDA reaction of the E-2-aryl-1-cyano-1-nitroethenes **1a**–**g** and methylenecyclopentane **2**.

The cycloaddition reactions between E-2-aryl-1-cyano-1-nitroethenes **1a**–**g** and methylenecyclopentane **2** theoretically can be realized according to two independent paths leading to regioisomeric HDA cycloadducts (Scheme 1).

Firstly we decided to shed light on the nature of global and local interactions between cycloaddition components. We analysed the electronic properties of E-2-aryl-1-cyano-1-nitroethenes in the Conceptual Density Functional Theory (CDFT) framework within our previous works [35]. It was found that all of these-type compounds exhibit an electrophilic nature. Subsequently, independently of the nature of the substituent at the 4-position of the phenyl ring, the most electrophilic centre is always located on the β-carbon atom of the nitrovinyl moiety. For the comparison, methylenecyclopentane **2** is characterized by a low value of global electrophilicity (Table 1). On the other hand, high global nucleophilicity is associated with this molecule. Consequently, role **2** in reactions with strong electrophilic nitroalkenes is rather clear. In the Domingo, ref. [36] terminology, considered processes can be treated as polar with reverse electron density flux (REDF). For these-type processes, the reaction regioselectivity can be predicted using local reactivity indices calculated in the framework of CDFT [37]. According to this approach, the reaction course is controlled by stabilized interactions between the most electrophilic centre of the first molecule with the most nucleophilic centre at the second one. In the case of considered reactions, it will be the nucleophilic attack of the C1 atom of methylenecyclopentane (Table 1 and Table 2) to the 2-position of the nitrovinyl moiety of nitroalkenes. These types of interactions favoured the formation of adducts **3**.

In our research’s next step, we attempted to verify the CDFT prediction. This study started from the reaction with the participation of parent E-2-phenyl-1-cyano-1-nitroethene **1c**. For this purpose, we examined a number of reaction samples using different solvents, reaction times, and reagents ratios. It was found that the reaction proceeded well in a chloroform solution at 60 °C with 10%mol excess of the alkene **2**. The reaction progress was monitored using chromatographic (TLC and HPLC) techniques. The cycloaddition was stopped after 24 h. Only one reaction product was detected in the post-reaction mixture. It was isolated via flash chromatography and crystallisation from ethyl acetate/petroleum ether or acetonitrile. In this way, we obtained the chemical individuum with the purity appropriate for identification. In particular, bands characteristic for C=N bonds were detected on the IR spectrum.

Next, we analysed their high-resolution mass spectra obtained by atmospheric pressure chemical ionization technique. The mass spectrum is characterized by protonated molecular ions in positive mode and deprotonated molecular ions in negative mode, appropriately, and a few prominent fragment ions. The elemental composition of the protonated molecular ion with *m*/*z* 257.1291 in positive mode and deprotonated molecular ion with *m*/*z* 255.1138 in negative mode confirmed the molecular formula C_15_H_16_N_2_O_2_. The major fragment ions in negative APCI mass spectra (*m*/*z* values and molecular formulas) are summarized in Table S1 (Supporting Information). Two of the fragmentation peaks, which correspond to the ions formed by the elimination of C_5_H_8_O and C_5_H_7_NO_2_, allowed us to assume that the cycloaddition reaction may lead to regioisomer **3c**. On the ^1^H NMR spectrum, the most important signal is observed at 4.01ppm as a doublet of doublets. This confirms postulated regioisomerism of the adduct obtained.

**Figure 1:**
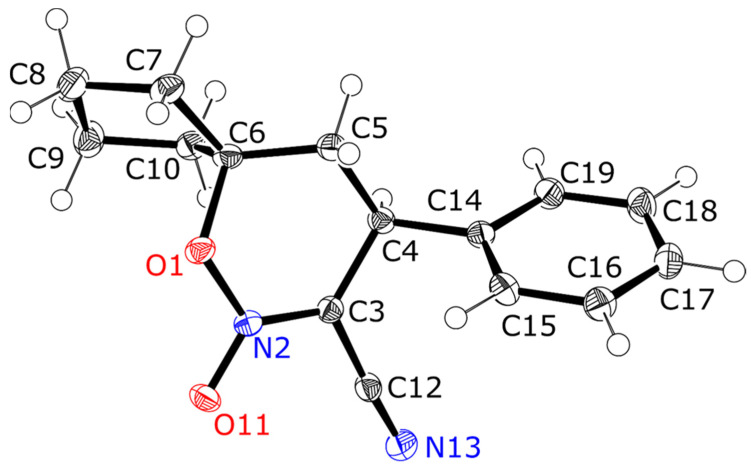
Molecular structure of 8-cyano-9-phenyl-6-oxa-7-aza-spiro-[4.5]dec-7-ene 7-oxide **3c**. Ellipsoids are shown at 50% probability.

**Figure 2:**
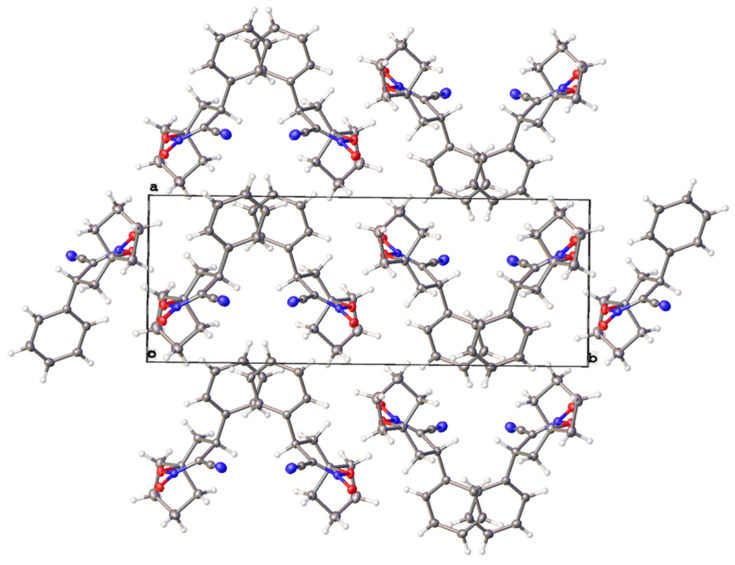
Crystal packing in 8-cyano-9-phenyl-6-oxa-7-aza-spiro-[4.5]dec-7-ene 7-oxide **3c** along the c axis.

Finally, the X-ray single crystal diffraction experiment revealed a molecular and crystal structure of 8-cyano-9-phenyl-6-oxa-7-aza-spiro-[4.5]dec-7-ene 7-oxide **3c**. The molecular geometry parameters are presented in the Supplementary Material. The compound crystallizes as colourless transparent prisms in a monoclinic P21/c space group. (Figure 1). The supramolecular structure is stabilized by hydrophobic interactions of phenyl and cyclopentane groups, while no hydrogen bonds or π-stacking were observed (Figure 2).

For a full description of the **1c+2** process, we decided additionally for deeper exploration of cycloaddition mechanism using DFT computational data. It should be underlined that the mechanism of the formation of the heterocyclic ring cannot be apriori assumed. Many known DA and HDA reactions are realized via a single-step mechanism [38]. In the last years, however, several examples of the reaction with the participation of zwitterionic [39,40,41] and biradical intermediates were discovered [42]. These cases were recently discussed critically [43,44]. For this purpose, the ωB97X-D/6-311G-(d) level of theory was applied. The similar approach we used previously for the discussion of mechanistic aspects of many bimolecular reactions, such as DA processes [45], [3 + 2] cycloadditions [46], [4 + 3] cycloaddition [47] and many other [35,48].

Analysis of the stationary points in the HDA reaction between E-2-aryl-1-cyano-1-nitroethenes **1a**–**g** and methylenecyclopentane **2** demonstrate that these reactions take place through a one-step mechanism. Relative enthalpies, Gibbs free energy and entropies of the stationary points are collected in Table 3. The reaction between **1c** and **2** begins with the creation of pre-reaction molecular complexes **MC_3c_** and **MC_4c_**. This stage is characterized by a reduction of the enthalpy of the reaction of about 6.1 and 7.7 kcal/mol, respectively (Table 3). Further conversion of MCs leads to TSs. This step is related to the growth of the enthalpy for **TS_3c_** and **TS_4c_** to 8.1 and 22.8 kcal/mol. The IRC analysis connects without any doubts both TSs, with respective MCs and respective products **3c** and **4c**. We can confirm that, in accordance with the experimental results, only one **3c** product was obtained without any trace of **4c** regioisomer.

**Figure 3:**
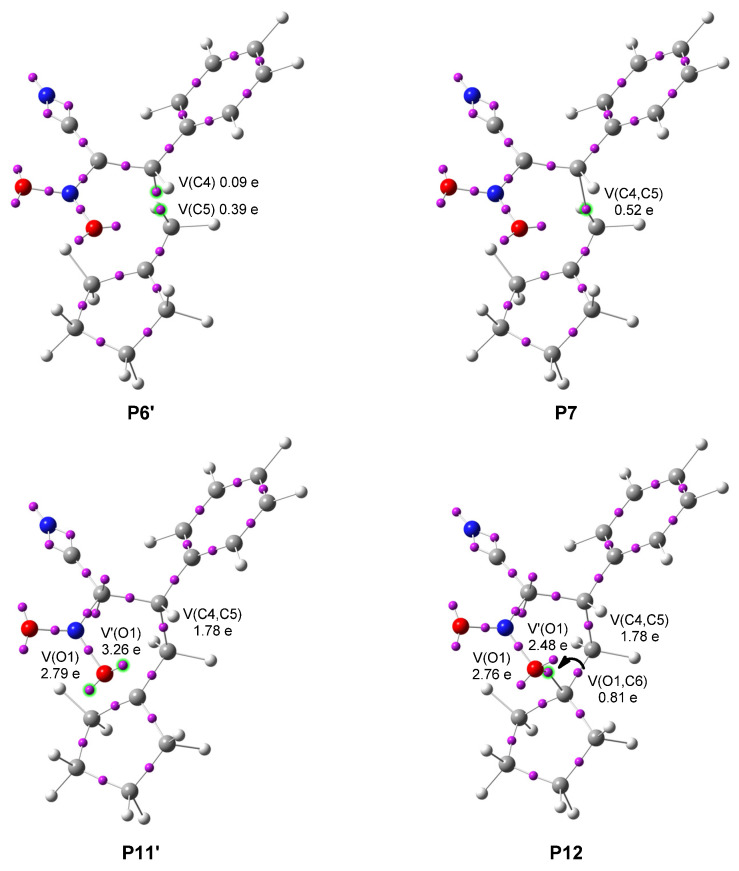
ELF attractor positions of the structures involved in the C-O and C-C bond formation on the reaction path of (E)-2-phenyl-1-cyano-1-nitroethene **1c** and alkene **2**. The prime symbol marks the last structure of the corresponding BET phase.

A bonding evolution theory (BET) study along the model reaction path of [4 + 2] cycloaddition of (E)-2-phenyl-1-cyano-1-nitroethene **1c** with methylenecyclopentane **2** was performed in order to characterize its mechanism. Detailed BET data of the important points of the reaction is given in Table 4 and Figure 3. The change in electronic structure, specifically the creation or disappearance of a basin compared to the previous point, is the choice’s condition.

**Scheme 2:**
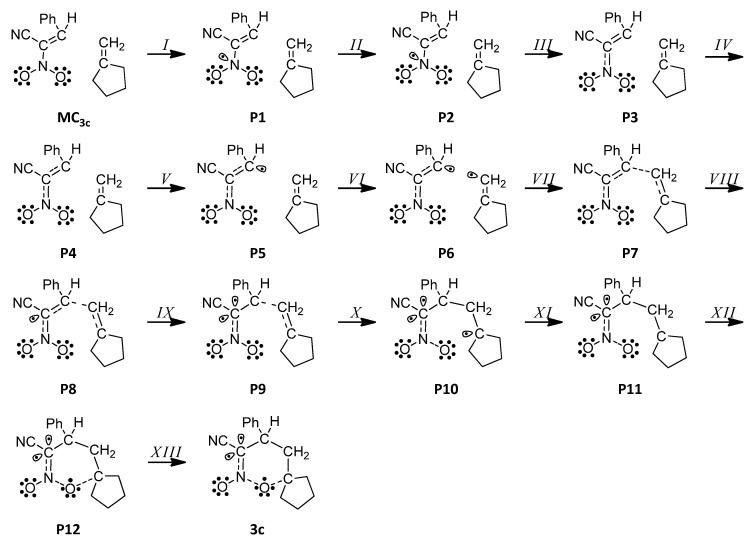
Simplified representation of the molecular mechanism of the HDA reaction between (E)-2-phenyl-1-cyano-1-nitroethene **1c** with methylenecyclopentane **2** by Lewis-like structures arising from the topological analysis of the ELF along the reaction path.

The BET study of 621 points along the reaction path revealed thirteen phases by following intrinsic reaction coordinates. The resulting observations can be noted:

(1) During phases, *I*–*V*, the topological changes heading to the creation of two *pseudoradical* centres [49] at C4 and C5 are taking place. At point **P1** V(N2) monosynaptic basin representing nonbonding electron density is created, integrating 0.05 e originating from disynaptic basins V(N2,O1) and V(N2,O11). At the next point two disynaptic basins V(C3,C4) and V’(C3,C4) of the double bond merges into one V(C3,C4) basin with a population of 3.51 e. The monosynaptic basin V(N2) disappears at point **P3** increasing V(N2,C3) disynaptic basins population to 2.51 e. Phase *V* starts with two disynaptic basins V(C5,C6) and V’(C5,C6) combining into one V(C5,C6) integrating 3.22 e. These changes contribute 11.2 kcal∙mol^−1^ of the energetic cost, in contrast, the transition state is only 3.29 kcal∙mol^−1^ higher.
(2) Phase *VI* begins at **P5** with the appearance of a *pseudoradical* center at C4 represented by a monosynaptic basin V(C4) with a population of 0.10 e gathered from the V(C4,C3) disynaptic basin. In the next phase *VII*, another *pseudoradical* centre is created at C5–the V(C5,C6) disynaptic basin provides electron density for a new monosynaptic basin V(C5) with initial integration of 0.36 e.
(3) The new bond C4-C5 is created at point **P7**, at a C-C distance of 2.000 Å by merging of V(C4) and V(C5) monosynaptic basins which show a population of 0.09 e and 0.39 e respectively at the last point of phase *VII*. The new disynaptic basin V(C4,C5) integrates 0.52 e, representing 28% of its final electron population. The **TS_3c_** is part of Phase *VIII*, where the first bond is already established.
(4) At points **P8** and **P9**, a new *pseudoradical* centre emerges represented by V(C3) and V’(C3) monosynaptic basins with an initial population of 0.52 e and 0.33 e, respectively. Their electron density originating from the V(C4,C3) disynaptic basin of the C4-C3 underpopulated double bond causes its transition into a single bond.
(5) At point **P10** the V(C6) monosynaptic basin appears with a marginal population of 0.01 e. At the start of phase *XII*, it disappears, and a slight increase in the population of V(O1) and V’(O1) monosynaptic basins takes place.
(6) At the beginning of the last phase, the second bond is created, at an O-C distance of 1.778 Å, by a donation of the nonbonding electron density of O1 to C6. The V(O1,C6) disynaptic basin integrates 0.81 e representing a strongly underpopulated O1-C6 single bond.
(7) The formation of the second O1-C6 bond starts while the first C4-C5 single bond is at 97% of its final population. Therefore, the mechanism of the HDA reaction of nitroalkene **1c** with alkene **2** proceeds by a two-stage one-step mechanism [50].
(8) The activation energy associated with this HDA reaction, 7.46 kcal·mol^−1^, can mainly be related to the depopulation of the C3-C4 and C5-C6 regions, formation of C4 and C5 pseudoradical centres and first C4-C5 single bond.
(9) The conducted BET analysis allowed for the determination and examination of the molecular mechanism of the HDA reaction of the (E)-2-phenyl-1-cyano-1-nitroethene **1c** and methylenecyclopentane **2** (Scheme 2).

In the last part of the research, we examined in our laboratory other similar processes with the participation of a series of E-2-aryl-1-cyano-1-nitroethenes with different-type EDG or EWG substituents in the 4 position of the phenyl ring. The results are collected in Table 5. All considered reactions are realized under similar conditions and with the same regioselectivity.

## 3. Materials and Methods

### 3.1. Analytical Techniques

HPLC analyses were done using a Knauer device with a UV VIS detector (LiChrospher 18-RP 10 µm column, eluent: 80% methanol). M.p. values were measured on the Boetius apparatus and were uncorrected. IR spectra were derived from the FTS Nicolet IS 10 spectrophotometer. ^1^HNMR spectra were recorded on an AV 400 Neo spectrometer or a Bruker Avance III 600 spectrometer and are reported in ppm using deuterated solvent as an internal standard (CDCl_3_ at 7.26 ppm).

### 3.2. X-ray Crystal Structure Determination

X-ray quality crystals of the 8-cyano-9-phenyl-6-oxa-7-aza-spiro-[4.5]dec-7-ene 7-oxide **3c**. were formed over a period of approximately 2 days by re-crystallization from ethanol. The diffraction intensities from the single crystal with dimensions of 0.7 × 0.6 × 0.4 mm were collected at T = 100.00(10) K with the use of Rigaku XtaLAB Synergy-S diffractometer equipped with a Cu Kα radiation source (λ = 1.54184 Å) and HyPix-6000HE hybrid photon counting detector. The total number of runs and images was based on the strategy calculation from the program CrysAlisPro (Rigaku, v1.171.41.123a, 2022), and the unit cell was refined using CrysAlisPro on 35,589 reflections, 74% of the observed reflections. The maximum resolution that was achieved was Θ = 75.837° (0.795 Å). The molecular model of the structure was obtained by the SHELXT [51] structure solution program using intrinsic phasing with Olex2 [52] as the graphical interface and refined by the least squares using version 2018/3 of SHELXL [53]. All non-hydrogen atoms were refined anisotropically. All of the hydrogen atom positions were calculated geometrically and refined in geometrically idealized positions with isotropic temperature factors 1.2 times the equivalent isotropic temperature factors, Ueq, of their attached atoms. The final structure was validated by CheckCif (http://checkcif.iucr.org, (accessed on 24 April 2023)) and deposited in the Cambridge Crystallographic Data Centre (CCDC) under accession number 2220704.

### 3.3. Synthesis of Conjugated Nitroalkenes

Nitroalkenes were prepared via condensation of appropriate aldehydes with nitroacetonitrile using known procedures [54,55,56]. 

### 3.4. Synthesis of Methylenecyclopentane (***2***)

2-Cyclopentenylacetonitrile [57]

Cyclopentanone (38.90 g, 0.4624 mol), α-cyanoacetic acid (39.33 g, 0.4624 mol), ammonium acetate (3.55 g, 0.0461 mol), and toluene (50 mL) was heated with magnetic stirring under reflux with a Dean-Stark adapter until no more water was separating (about 4 h). Next, the reaction mixture was cooled to room temperature and washed twice with 25 mL of water, once with 25 mL of a saturated aqueous solution of NaHCO_3_, and twice with 25 mL of brine. The solvent was removed on a rotary evaporator and the residue was distilled under reduced pressure to give 43.31 g (0.4041 mol) of 2-cyclopentenylacetonitrile with a boiling point of 102 106 °C/70 mmHg which corresponds to a yield of 87.4%.

2-Cyclopentenylacetic acid

2-Cyclopentenylacetonitrile (40.00 g, 0.3733 mol), ethylene glycol (100 mL), and sodium hydroxide (45 g, 1.125 mol) solution in water (100 mL) was heated under reflux for 15 h. After that time the reaction mixture was cooled to room temperature, diluted with water (200 mL) neutralized with con. hydrochloric acid (100 mL), and extracted with five portions of dichloromethane (25 mL). The organic phase was washed twice with water (100 mL), dried with anhydrous MgSO_4_, and evaporated on a rotary evaporator. Obtained crude 2-cyclopentenylacetic acid (38.72 g, 0.3068 mol, 82.2%) was used for decarboxylation without further purification.

Methylenecyclopentane (**2**)

2-Cyclopentenylacetic acid (38.72 g, 0.3069 mol) was charged into a 100 mL round bottom flask, connected to a distillation apparatus with a receiver cooled in an isopropanol/liquid nitrogen slush bath (−80 °C) and was slowly heated in an oil bath. When the oil bath reached 145 °C a reaction started. Distillate was collected at a temperature not exceeding 80 °C. When no more liquid was condensing the distillation was stopped and the crude product was washed with Na_2_CO_3_ (20 mL of 10% aqueous solution), and dried with anhydrous CaCl_2_. Fractional distillation gave 14.29 g (0.1740 mol, 56.7%) of methylenecyclopentane with a boiling point of 71–73 °C.

### 3.5. HDA Reactions between E-2-Aryl-1-cyano-1-nitroethenes and Methylenecyclopentane–General Procedure

A glass ampule was charged with 0.002 mol of nitroalkene **1**, 0.0022 mol (0.1809 g) of methylenecyclopentane **2**, and chloroform (5 mL). After sealing, the ampule was heated in a water bath at 60 °C for 24 h. Next, the ampule was cooled in an ice bath and opened. The crude reaction mixture was evaporated on a rotary evaporator. The remaining solid was subjected to flash chromatography and crystallization from ethyl acetate/petroleum ether or acetonitrile.

### 3.6. Quantum Chemical Calculations

DFT calculations were performed using GAUSSIAN 16 package [58]. Frequency analysis was used to characterize all stationary points; substrates and products presented only positive eigenvalues in their Hessian matrices and transition states (TSs) presented only one negative eigenvalue in their Hessian matrices. The solvent effects on the reaction were included by using the IEFPCM algorithm [59]. All calculations were done for molecules at 298.15 K and 1 atm. 

The global electron density transfer [60] (GEDT) was calculated using the equation GEDT $\left(f\right)=\underset{q\in f}{\sum }q$, where *q* are the charges, computed by natural population analysis [61,62] (NPA), of all atoms belonging to one of the two frameworks (*f*) at the TS. The global reactivity indices (electronic potential μ, chemical hardness η, global electrophilicity ω, and global nucleophilicity N) were calculated at B3LYP/6-31G(d) computational level using the equation described in ref. [63].

The Electron Localization Function (ELF) [64] analysis was performed using the TopMod package [65] at the standard cubical grid of step size of 0.1 Bohr. To visualize the molecular geometries and ELF basin attractors GaussView program [66] was used.

## 4. Conclusions

HDA reactions with the participation of E-2-aryl-1-cyano-1-nitroethenes **1a**–**g** and methylenecyclopentane **2** are realised very easily under mild conditions. In contrast to most known nitroalkene HDA processes, the explored reaction does not require the presence of LA-type catalysts. Additionally, in all analysed cases, the full regioselectivity of the reaction was observed. So, the synthetic protocol proposed by us should be considered very attractive for the comparison of a generally known standard. Next, our theoretical investigations show, without any doubts, that the reaction regiocontrol can be easily predicted in the framework of CDFT. Additionally, performed mechanistic considerations confirm that the process of the formation of a new heterocyclic molecular system is realized via polar but one-step mechanism. Detailed BET analysis of the reaction between (E)-2-phenyl-1-cyano-1-nitroethene **1c** with methylenecyclopentane **2** indicates that at the beginning of the reaction, two pseudoradical C4 and C5 centres are formed, demanded the formation of the first C4-C5 single bond through a two-centre interaction. Formation of the second O1-C6 bond takes place at the end of the reaction path when the first C4-C5 single bond is almost formed. According to that, the mechanism of HDA reaction follows a *two-stage one-step* mechanism.

## Figures and Tables

**Table 1:** Global and local electronic properties of methylenecyclopentane **2**.

Global Properties				Local Properties			
eletronic chemical potential μ [eV]	chemical hardhess η [eV]	electro- philicity ω [eV]	nucleo- philicityN [eV]	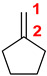			
				P^−^_1_	N_1_ [eV]	P^−^_2_	N_2_ [eV]
2.87	6.95	0.59	2.77	0.636	1.76	0.266	0.74

**Table 2:** The global reactivity descriptors of E-2-aryl-1-cyano-1-nitroethenes **1a**–**g** are calculated at the B3LYP/6-31G(d) level of theory. The electronic chemical potential μ, chemical hardness η, electrophilicity ω, and nucleophilicity N of studied reagents in eV.

Nitroalkene	Eletronic Chemical Potential [μ eV]	Chemical Hardhess η [eV]	Electro- Philicity [eV]	Nucleo- Philicityn [eV]
**1a**	−4.82	3.69	3.14	2.45
**1b**	−5.10	3.94	3.30	2.04
**1c**	−5.27	4.06	3.42	1.80
**1d**	−5.28	3.98	3.50	1.84
**1e**	−5.35	3.89	3.67	1.81
**1f**	−5.31	3.81	3.70	1.90
**1g**	−5.49	3.96	3.80	1.64

**Table 3:** ωB97X-D (PCM)/6-311 G(d) thermodynamic parameters: Gibbs free energy, enthalpies in kcal∙mol-1 and entropies in kcal∙mol^−1^∙K^−1^ calculated at 25 °C and 1atm in chloroform. Imaginary frequencies characteristic for transition states in cm^−1^.

Structure	ΔH	ΔS	ΔG	Imaginary Frequencies
**MC_3a_**	–6.1	–32.6	3.7	-
**TS_3a_**	10.0	–46.6	23.9	−425.7
**3a**	–22.2	–50.6	–7.1	-
**MC_4a_**	–7.8	–37.5	3.4	-
**TS_4a_**	24.1	–53.1	39.9	−586.6
**4a**	–15.8	–52.4	–0.1	-
**MC_3b_**	–6.1	–33.4	3.8	-
**TS_3b_**	8.3	–52.9	24.1	−420.3
**3b**	–23.8	–50.5	–8.7	-
**MC_4b_**	–7.9	–37.3	3.2	-
**TS_4b_**	23.2	–49.9	38.1	−581.7
**4b**	–17.3	–51.9	–1.8	-
**MC_3c_**	–6.1	–34.0	4.0	-
**TS_3c_**	8.1	–46.4	21.9	−421.4
**3c**	–24.7	–50.1	–9.8	-
**MC_4c_**	–7.7	–37.0	3.3	-
**TS_4c_**	22.8	–53.6	38.7	−579.5
**4c**	–18.3	–53.5	–2.3	-
**MC_3d_**	–6.1	–33.8	3.9	-
**TS_3d_**	8.4	–47.3	22.6	−422.5
**3d**	–24.5	–50.3	–9.5	-
**MC_4d_**	–7.8	–36.6	3.2	-
**TS_4d_**	22.9	–52.8	38.7	−580.3
**4d**	–18.0	–52.3	–2.4	-
**MC_3e_**	–6.1	–34.6	4.2	-
**TS_3e_**	7.9	–47.7	22.1	−422.2
**3e**	–25.3	–51.0	–10.1	-
**MC_4e_**	–7.9	–38.6	3.6	-
**TS_4e_**	22.5	–53.2	38.3	−578.9
**4e**	–18.8	–53.0	–3.0	-
**MC_3f_**	–6.1	–36.4	4.8	-
**TS_3f_**	7.9	–49.0	22.5	−421.7
**3f**	–25.3	–52.2	–9.7	-
**MC_4f_**	–7.9	–38.0	3.5	-
**TS_4f_**	22.4	–53.5	38.3	−579.3
**4f**	–18.9	–54.0	–2.8	-
**MC_3g_**	–6.3	–33.7	3.8	-
**TS_3g_**	7.0	–46.2	20.8	−421.5
**3g**	–26.2	–50.9	–11.0	-
**MC_4g_**	–8.0	–37.9	3.3	-
**TS_4g_**	22.0	–52.1	37.5	−578.0
**4g**	–19.7	–53.0	–3.9	-

**Table 4:** ELF valence basin populations, distances of the rupture and forming bonds and relative electronic energies of the IRC points, **MC_3c_**–**3c**, defining the thirteen different phases characterizing the reaction of the E-2-phenyl-1-cyano-1-nitroethene **1c** and methylenecyclopentane **2**. The stationary points **1c**, **2**, **MC_3c_**, **TS_3c_** and **3c** are also included. Distances are given in angstroms, Å, electron populations in an average number of electrons, [e], relative energies in kcal·mol^−1^, GEDT values in an average number of electrons, [e] and IRC values in a.u.

Structures	2	1c	MC_3c_		P1		P2		P3		P4		P5		P6		P7	TS_3c_	P8		P9		P10		P11		P12		3c
Phases				I		II		III		IV		V		VI		VII		VIII		IX		X		XI		XII		XIII	
d1(C1-C14)			3.131		3.126		2.861		2.405		2.240		2.039		2.019		2.000	1.962	1.954		1.896		1.563		1.557		1.539		1.543
d2(O9-C15)			3.467		3.454		3.189		2.946		2.896		2.850		2.846		2.842	2.834	2.831		2.818		2.098		2.022		1.778		1.478
IRC			−9.67		−9.25		−5.43		−2.25		−1.38		−0.39		−0.29		−0.19	0.00	0.03		0.32		2.31		5.98		7.39		
GEDT			0.01		0.01		0.03		0.11		0.20		0.36		0.38		0.40	0.42	0.44		0.50		0.61		0.59		0.52		
dE kcal			−7.03		−6.98		−5.35		0.96		4.17		7.15		7.29		7.38	7.46	7.46		7.25		−3.66		−5.61		−13.70		−23.48
V(O1)		2.83	2.84		2.83		2.84		2.86		2.85		2.87		2.87		2.87	2.88	2.88		2.89		2.96		2.88		2.48		2.60
V’(O1)		2.80	2.82		2.82		2.82		2.83		2.88		2.90		2.91		2.91	2.91	2.91		2.93		2.91		3.05		2.76		2.39
V(N2.O1)		1.85	1.85		1.88		1.88		1.81		1.77		1.71		1.71		1.70	1.69	1.69		1.67		1.41		1.39		1.30		1.23
V(N2.O11)		2.19	2.19		2.10		2.07		2.03		1.94		1.84		1.83		1.83	1.82	1.81		1.79		1.67		1.65		1.64		1.65
V(C3.N2)		2.29	2.31		2.32		2.32		2.51		2.62		2.76		2.77		2.78	2.80	2.80		2.84		3.29		3.35		3.50		3.60
V(C4.C3)		1.76	1.66		1.66		3.51		3.50		3.49		3.42		3.41		3.39	3.36	2.83		2.42		2.06		2.05		2.04		2.04
V’(C4.C3)		1.79	1.86		1.87																								
V(C5.C6)	1.78		1.73		1.73		1.78		1.77		3.28		3.22		2.89		2.85	2.77	2.76		2.65		2.17		2.14		2.07		2.01
V’(C5.C6)	1.78		1.79		1.79		1.70		1.61																				
V(O1.C6)																											0.81		1.22
V(C4.C5)																	0.52	0.65	0.67		0.84		1.70		1.72		1.78		1.83
V(N2)					0.05		0.08																						
V(C3)																			0.52		0.56		0.57		0.55		0.49		0.37
V’(C3)																					0.33		0.32		0.31		0.26		0.30
V(C4)													0.10		0.09														
V(C5)															0.36														
V(C6)																							0.01						

**Table 5:** Selected conditions for the HDA reaction of the E-2-aryl-1-cyano-1-nitroethenes **1a**–**g** and methylenecyclopentane **2**.

Reaction	Solvent	Temperature [°C]	Time [h]	Yield [^%^]
**1a+2**	Chloroform	60	24	22
**1b+2**	Chloroform	60	24	32
**1c+2**	Chloroform	0	24	-
**1c+2**	Chloroform	25	24	trace
**1c+2**	Chloroform	60	12	40
**1c+2**	Chloroform	60	24	58
**1c+2**	Nitromethane	60	24	52
**1d+2**	Chloroform	60	24	61
**1e+2**	Chloroform	60	24	60
**1f+2**	Chloroform	60	24	72
**1g+2**	Chloroform	60	24	69

## Data Availability

Not applicable.

## Supplementary Materials

The following supporting information can be downloaded at: https://www.mdpi.com/article/10.3390/molecules28124586/s1.
