# Supplementary material for: Fully Selective Synthesis of Spirocyclic-1,2-oxazine N-Oxides via Non-Catalysed Hetero Diels-Alder Reactions with the Participation of Cyanofunctionalysed Conjugated Nitroalkenes
Source: Molecules. 2023 Jun 6;28(12):4586. doi: 10.3390/molecules28124586 (PMC10301638; doi:10.3390/molecules28124586)
Supplement: Supplementary file 1 [file molecules-28-04586-s001.zip › molecules-2441140-supplementary.pdf]

Supplementary Material

Fully Selective Synthesis of Spirocyclic-1,2-oxazine N-Oxides via Non-Catalysed Hetero Diels-Alder Reactions with the Participation of Cyanofunctionalysed Conjugated Nitroal-kenes

Przemysław Woliński, Agnieszka Kącka-Zych, Aneta Wróblewska,
Ewelina Wielgus, Rafał Dolot, Radomir Jasiński

[1] Analytical techniques 2

[2] X-ray crystal structure determination . . . 3

[3] Spectral characteristics 4

[1] Analytical techniques

^{1}HNMR spectra were recorded on a AV 400 Neo spectrometer and are reported in ppm using deuterated solvent as an internal standard ($CDCl_3$ at 7.26 ppm). Data are reported as s = singlet, dd = doublet of doublets, m = multiplet. ^{13}CNMR spectra were recorded on a AV 400 Neo 101 MHz spectrometer and are reported in ppm using deuterated solvent as an internal standard ($CDCl_3$ at 77.23 ppm). ^{19}FNMR spectrum was recorded on a AV 400 Neo 376 MHz spectrometer, and reported in ppm using deuterated solvent as an internal standard $CDCl_3$.

High-resolution mass spectrometry (HRMS) measurements were performed using Synapt G2-S*i* mass spectrometer (Waters, Milford, MA, USA) equipped with an atmospheric pressure chemical ionization (APCI) source and quadrupole Time-of-Flight mass analyzer. The mass spectrometer was operated in the positive or negative ion detection mode with a discharge current set at 4.0 µA. The heated capillary temperature was 350 °C. To ensure accurate mass measurements, data were collected in centroid mode and mass was corrected during acquisition using leucine enkephalin solution as an external reference (Lock-SprayTM), which generated reference ion at *m/z* 556.2771 Da ($[M + H]^+$) in positive mode and at *m/z* 554.2615 Da ($[M - H]^-$) in negative mode. The results of the measurements were processed using MassLynx 4.1 software (Waters) incorporated with the instrument.

HPLC analyses was done using a Knauer device with a UV VIS detector (LiChrospher 18-RP 10 µm column, eluent: 80% methanol). M.p. values were measured on the Boetius apparatus and were uncorrected. IR spectra were derived from the FTS Nicolet IS 10 spectrophotometer.

[2] X-ray crystal structure determination

X-ray quality crystals of the 8-cyano-9-phenyl-6-oxa-7-aza-spiro-[4.5]dec-7-ene 7-oxide **3c**. were formed by re-crystallization from ethanol in a room temperature. The diffraction intensities from the single crystal with dimension of 0.7 × 0.6 × 0.4 mm were collected at T = 100.00(10) K with the use of Rigaku XtaLAB Synergy-S diffractometer equipped with a Cu Kα radiation source (λ = 1.54184 Å) and HyPix-6000HE hybrid photon counting detector. The total number of runs and images was based on the strategy calculation from the program *CrysAlisPro* (Rigaku, v1.171.41.123a, 2022) and the unit cell was refined using *CrysAlisPro* on 35589 reflections, 74% of the observed reflections. The maximum resolution that was achieved was Θ = 75.837° (0.795 Å). The molecular model of the structure was obtained by the *SHELXT* structure solution program using intrinsic phasing with *Olex2* as the graphical interface and refined by the least squares using version 2018/3 of *SHELXL*. All non-hydrogen atoms were refined anisotropically. All of hydrogen atom positions were calculated geometrically and refined in geometrically idealized positions with isotropic temperature factors 1.2 times the equivalent isotropic temperature factors, U_{eq}, of their attached atoms. The final structure was validated by CheckCif (http://checkcif.iucr.org) and deposited in the Cambridge Crystallographic Data Centre (CCDC) under accession number 2220704.

[3] Spectral characteristics

8-cyano-9-(-4-methoxy-phenyl)-6-oxa-7-aza-spiro-[4.5]dec-7-ene 7-oxide **3a**

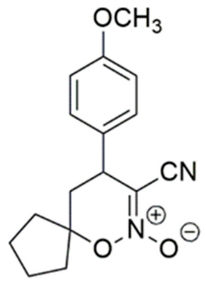

mp: 133–134 °C (acetonitrile)

TLC: Rf 0.21 (ethyl acetate/ petroleum ether, 25/75, UV)

^{1}HNMR (400 MHz, $CDCl_3$): δ 7.23–7.11 (m, 2H), 7.01–6.86 (m, 2H), 3.96 (dd, $J_{H\text{-}H}$ = 10.6 Hz, $J_{H\text{-}H}$ = 7.9 Hz, 1H), 3.83 (s, 3H), 2.30–2.13 (m, 3H), 2.12–2.03 (m, 1H), 2.01–1.89 (m, 2H), 1.87–1.65 (m, 4H) ppm.

^{13}CNMR (101 MHz, $CDCl_3$): δ 129.5, 129.0, 115.0, 112.6, 103.5, 97.2, 55.5, 41.0, 38.3, 38.1, 35.1, 24.6, 23.9 ppm.

FTIR ATR: 3020, 2965, 2938, 2920, 2875, 2839, 2289, 2210, 2073, 2040, 1981, 1889, 1766, 1617, 1587, 1549, 1516, 1462, 1447, 1438, 1431, 1332, 1320, 1308, 1293, 1268, 1233, 1203, 1176, 1135, 1117, 1103, 1047, 1024, 1011, 998, 972, 911, 891, 867, 828, 771, 733, 716, 679, 650, 630, 590, 554, 523, 512, 490, 457, 446, 428

HRMS (+APCI): *m/z* calcd for $C_{16}H_{19}N_2O_3$: 287. 1396 $[M + H]^+$; found 287.1401.

HRMS (−APCI): *m/z* calcd for $C_{16}H_{17}N_2O_3$: 285.1239 $[M + H]^-$; found 285.1237.

8-cyano-9-(-4-methyl-phenyl)-6-oxa-7-aza-spiro-[4.5]dec-7-ene 7-oxide **3b**

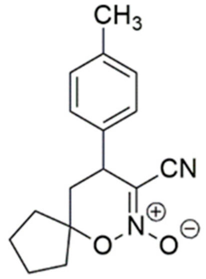

mp: 126–127 °C (acetonitrile)

TLC: Rf 0.31 (ethyl acetate/ petroleum ether, 25/75, UV)

^{1}HNMR (400 MHz, $CDCl_3$): δ 7.24–7.19 (m, 2H), 7.18–7.13 (m, 2H), 3.97 (dd, $J_{H\text{-}H}$ = 10.4 Hz, $J_{H\text{-}H}$ = 8.2 Hz, 1H), 2.37 (s, 3H), 2.78–2.12 (m, 3H), 2.11–2.02 (m, 1H), 2.00–1.89 (m, 2H), 1.88–1.67 (m, 4H) ppm.

^{13}CNMR (101 MHz, $CDCl_3$): δ 138.6, 134.6, 130.3, 127.6, 112.6, 103.4, 97.3, 41.4, 38.2, 38.0, 35.1, 24.6, 23.9, 21.3 ppm.

FTIR ATR: 3809, 3023, 2968, 2945, 2877, 2286, 2218, 2168, 2112, 1980, 1906, 1803, 1658, 1580, 1544, 1513, 1447, 1435, 1374, 1344, 1321, 1309, 1292, 1259, 1229, 1198, 1183, 1171, 1133, 1111, 1099, 1035, 1020, 996, 976, 951, 910, 888, 865, 823, 813, 783, 754, 729, 677, 650, 634, 556, 540, 514, 495, 484, 445, 435

HRMS (+APCI): *m/z* calcd for $C_{16}H_{19}N_2O_2$: 271. 1447 $[M + H]^+$; found 271.1446.

HRMS (−APCI): *m/z* calcd for $C_{16}H_{17}N_2O_2$: 269.1290 $[M + H]^-$; found 269.1291.

8-cyano-9-phenyl-6-oxa-7-aza-spiro-[4.5]dec-7-ene 7-oxide **3c**

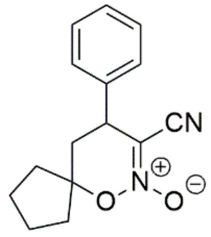

mp: 126–127 °C (ethyl acetate/petroleum ether)

TLC: Rf 0.27 (ethyl acetate/ petroleum ether, 25/75, UV)

^{1}HNMR (400 MHz, $CDCl_3$): δ 7.48–7.33 (m, 3H), 7.30–7.23 (m, 2H), 4.01 (dd, $J_{H\text{-}H}$ = 10.1 Hz, $J_{H\text{-}H}$ = 8.4 Hz, 1H), 2.30–2.23 (m, 2H), 2.22–2.14 (m, 1H), 2.12–2.04 (m, 1H), 2.02–1.90 (m, 2H), 1.87–1.67 (m, 4H) ppm.

^{13}CNMR (101 MHz, $CDCl_3$): δ 137.8, 129.7, 128.8, 127.8, 112.5, 103.1, 97.3, 41.8, 38.2, 38.1, 35.1, 24.6, 23.9 ppm.

FTIR ATR: 3775, 3073, 2975, 2881, 2285, 2213, 2162, 2050, 2038, 1954, 1884, 1814, 1760, 1588, 1562, 1498, 1457, 1446, 1428, 1350, 1325, 1294, 1256, 1234, 1196, 1180, 1161, 1141, 1115, 1078, 1049, 1033, 996, 973, 949, 916, 889, 871, 858, 760, 720, 699, 650, 629, 618, 563, 520, 498, 490, 441, 425

HRMS (+APCI): *m/z* calcd for $C_{15}H_{17}N_2O_2$: 257.1290 $[M + H]^+$; found 257.1291.

HRMS (−APCI): *m/z* calcd for $C_{15}H_{15}N_2O_2$: 255.1134 $[M + H]^-$; found 255.1138.

8-cyano-9-(-4-fluoro-phenyl)-6-oxa-7-aza-spiro-[4.5]dec-7-ene 7-oxide **3d**

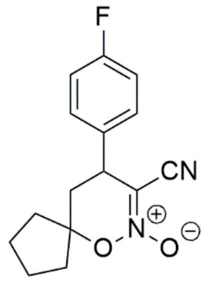

mp: 136–137 °C (ethyl acetate/petroleum ether)

TLC: Rf 0.23 (ethyl acetate/ petroleum ether, 25/75, UV)

^{1}HNMR (400 MHz, $CDCl_3$): δ 7.33–7.20 (m, 2H), 7.17–7.03 (m, 2H), 4.09–3.93 (m, 1H), 2.28–2.22 (m, 2H), 2.21–2.13 (m, 1H), 2.12–2.03 (m, 1H), 2.01–1.90 (m, 2H), 1.88–1.67 (m, 4H) ppm.

^{13}CNMR (101 MHz, $CDCl_3$): δ 162.8 (d, $J_{C\text{-}F}$ = 253.5 Hz), 133.5 (d, $J_{C\text{-}F}$ = 2.9 Hz), 129.5 (d, $J_{C\text{-}F}$ = 8.6 Hz), 116.7 (d, $J_{C\text{-}F}$ = 21.5 Hz), 112.5, 102.9, 97.3, 41.1, 38.2, 38.1 ppm.

^{19}FNMR (376 MHz, $CDCl_3$): δ -112.14 ppm.

FTIR ATR: 3753, 3677, 3657, 3630, 3071, 2957, 2877, 2219, 2166, 2112, 2036, 2028, 2014, 1979, 1907, 1783, 1664, 1607, 1561, 1509, 1469, 1451, 1438, 1426, 1349, 1329, 1308, 1289, 1254, 1234, 1220, 1195, 1164, 1140, 1115, 1098, 1045, 1032, 1019, 998, 973, 950, 913, 889, 862, 842, 819, 735, 685, 649, 629, 607, 565, 537, 517, 509, 489, 449, 426, 410

HRMS (+APCI): *m/z* calcd for $C_{15}H_{16}F_1N_2O_2$: 275.1196 $[M + H]^+$; found 275.1202.

HRMS (−APCI): *m/z* calcd for $C_{15}H_{14}F_1N_2O_2$: 273.1039 $[M + H]^-$; found 273.1044.

8-cyano-9-(-4-chloro-phenyl)-6-oxa-7-aza-spiro-[4.5]dec-7-ene 7-oxide **3e**

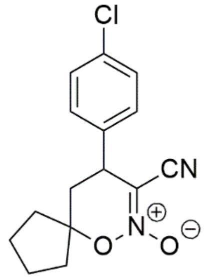

mp: 138–139 °C (acetonitrile)

TLC: Rf 0.25 (ethyl acetate/ petroleum ether, 25/75, UV)

^{1}HNMR (400 MHz, $CDCl_3$): δ 7.42–7.36 (m, 2H), 7.25–7.19 (m, 2H), 4.08–3.92 (m, 1H), 2.28–2.21 (m, 2H), 2.19–2.13 (m, 1H), 2.12–2.03 (m, 1H), 2.01–1.89 (m, 2H), 1.88–1.67 (m, 4H) ppm.

^{13}CNMR (101 MHz, $CDCl_3$): δ 136.3, 134.7, 129.9, 129.2, 112.4, 102.6, 97.3, 41.2, 38.2, 37.9, 35.1, 24.6, 23.9 ppm.

FTIR ATR: 3808, 3651, 3068, 2967, 2940, 2879, 2850, 2287, 2213, 2161, 2050, 1980, 1911, 1795, 1656, 1558, 1492, 1442, 1414, 1357, 1346, 1328, 1306, 1284, 1234, 1194, 1137, 1113, 1090, 1045, 1016, 997, 975, 946, 910, 891, 874, 862, 836, 824, 735, 706, 666, 648, 627, 561, 524, 502, 461, 434

HRMS (+APCI): *m/z* calcd for $C_{15}H_{16}Cl_1N_2O_2$: 291.0900 $[M + H]^+$; found 291.0902.

HRMS (−APCI): *m/z* calcd for $C_{15}H_{14}Cl_1N_2O_2$: 289.0746 $[M + H]^-$; found 289.0744.

8-cyano-9-(-4-bromo-phenyl)-6-oxa-7-aza-spiro-[4.5]dec-7-ene 7-oxide **3f**

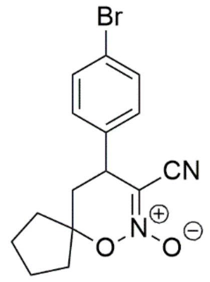

mp: 143–144 °C (acetonitrile)

TLC: Rf 0.25 (ethyl acetate/ petroleum ether, 25/75, UV)

^{1}HNMR (400 MHz, $CDCl_3$): δ 7.62–7.49 (m, 2H), 7.24–7.08 (m, 2H), 4.07–3.93 (m, 1H), 2.27–2.13 (m, 3H), 2.12–2.04 (m, 1H), 2.01–1.90 (m, 2H), 1.88–1.67 (m, 4H) ppm.

^{13}CNMR (101 MHz, $CDCl_3$): δ 136.8, 132.9, 129.5, 122.9, 112.4, 102.4, 97.3, 41.3, 38.3, 37.9, 35.2, 24.6, 24.0 ppm.

FTIR ATR: 3058, 3041, 3022, 2972, 2932, 2877, 2219, 2161, 2049, 1980, 1948, 1811, 1740, 1580, 1568, 1486, 1450, 1438, 1430, 1409, 1343, 1330, 1303, 1287, 1240, 1192, 1167, 1133, 1107, 1092, 1070, 1052, 1025, 1008, 1000, 973, 953, 912, 900, 869, 848, 820, 767, 732, 688, 646, 624, 571, 550, 520, 497, 449, 435, 406

HRMS (+APCI): *m/z* calcd for $C_{15}H_{16}Br_1N_2O_2$: 335.0398 $[M + H]^+$; found 335.0398.

HRMS (−APCI): *m/z* calcd for $C_{15}H_{14}Br_1N_2O_2$: 333.0239 $[M + H]^-$; found 333.0233.

8-cyano-9-(-4-methoxycarbonyl-phenyl)-6-oxa-7-aza-spiro-[4.5]dec-7-ene 7-oxide **3g**

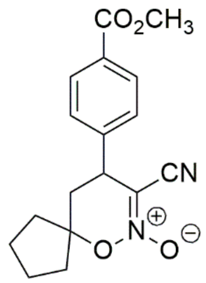

mp: 140–141 °C (acetonitrile)

TLC: Rf 0.15 (ethyl acetate/ petroleum ether, 25/75, UV)

^{1}HNMR (400 MHz, $CDCl_3$): δ 8.13–8.02 (m, 2H), 7.42–7.31 (m, 2H), 4.17–4.03 (m, 1H), 3.93 (s, 3H), 2.29–2.23 (m, 2H), 2.22–2.12 (m, 1H), 2.11–2.02 (m, 1H), 2.00–1.88 (m, 2H), 1.85–1.66 (m, 4H) ppm.

^{13}CNMR (101 MHz, $CDCl_3$): δ 166.5, 142.8, 130.9, 130.6, 127.9, 112.4, 102.3, 97.3, 52.4, 41.6, 38.2, 37.8, 35.1, 24.6, 23.9 ppm.

FTIR ATR: 2979, 2948, 2875, 2323, 2223, 2167, 2110, 1979, 1945, 1718, 1613, 1561, 1511, 1448, 1432, 1418, 1367, 1350, 1314, 1288, 1250, 1232, 1192, 1182, 1144, 1117, 1049, 1019, 1001, 964, 917, 888, 868, 859, 842, 831, 777, 717, 643, 633, 561, 523, 501, 485, 451, 429

HRMS (+APCI): *m/z* calcd for $C_{17}H_{19}N_2O_4$: 315.1345 $[M + H]^+$; found 315.1344.

HRMS (−APCI): *m/z* calcd for $C_{17}H_{17}N_2O_4$: 313.1188 $[M + H]^-$; found 313.1187.

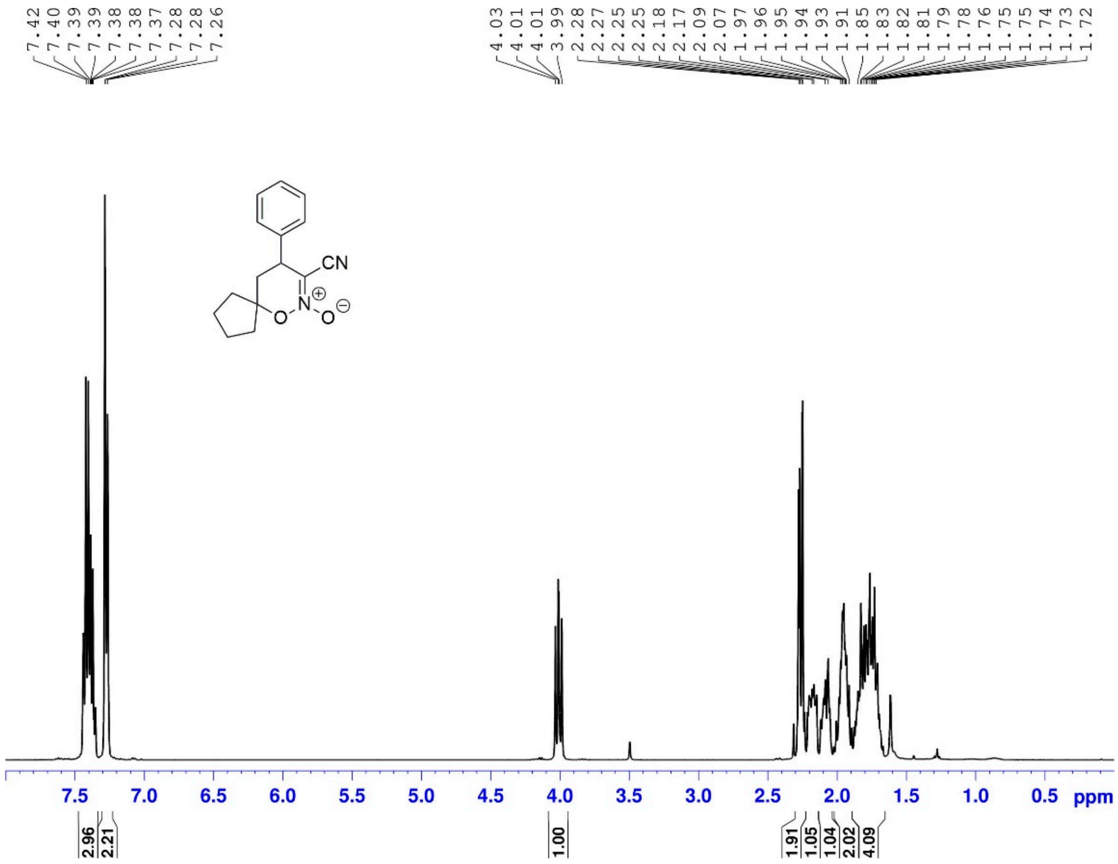
7.42 7.40 7.39 7.39 7.38 7.38 7.37 7.28 7.28 7.26
4.03 4.01 4.01 3.99 2.28 2.27 2.25 2.25 2.18 2.17 2.09 2.07 1.97 1.96 1.95 1.94 1.93 1.91 1.85 1.83 1.82 1.81 1.79 1.78 1.76 1.75 1.75 1.74 1.73 1.72
CN
N
O
O
7.5 7.0 6.5 6.0 5.5 5.0 4.5 4.0 3.5 3.0 2.5 2.0 1.5 1.0 0.5 ppm
2.96 2.21 1.00 1.91 1.05 1.04 2.02 4.09

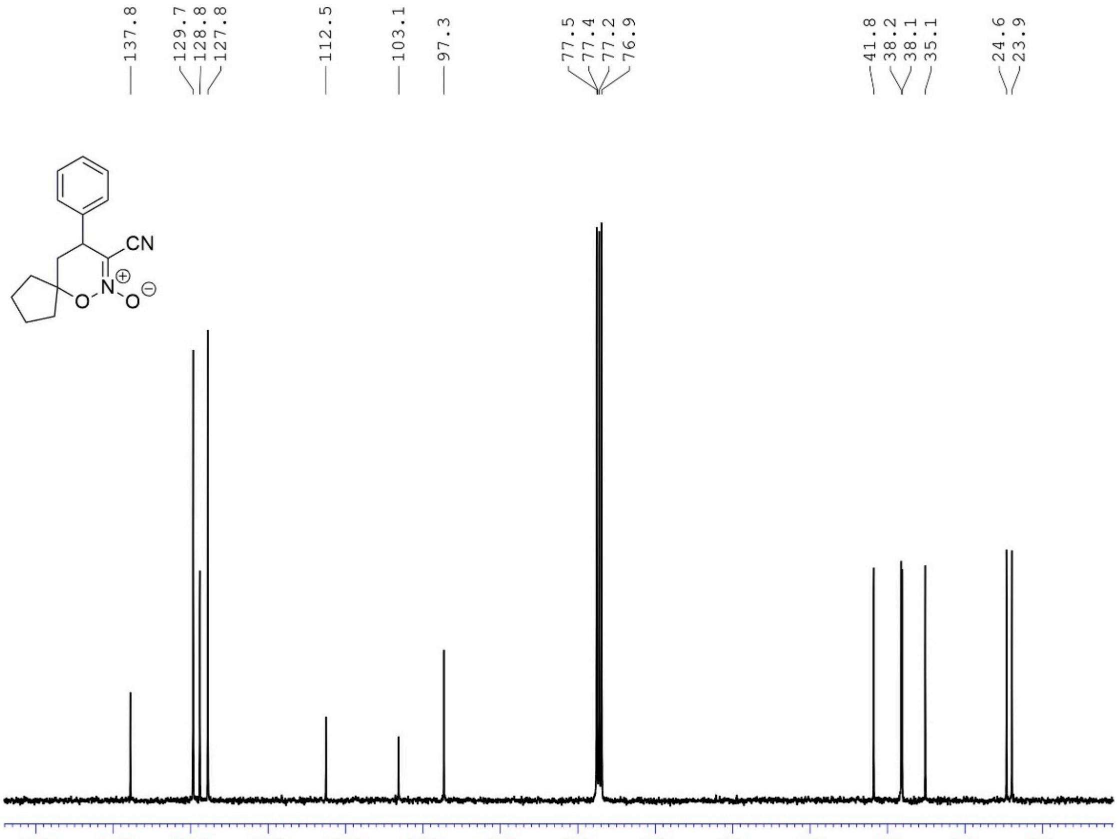
137.8 129.7 128.8 127.8 112.5 103.1 97.3 77.5 77.4 77.2 76.9 41.8 38.2 38.1 35.1 24.6 23.9
CN
N
O
O
150 140 130 120 110 100 90 80 70 60 50 40 30 20 ppm

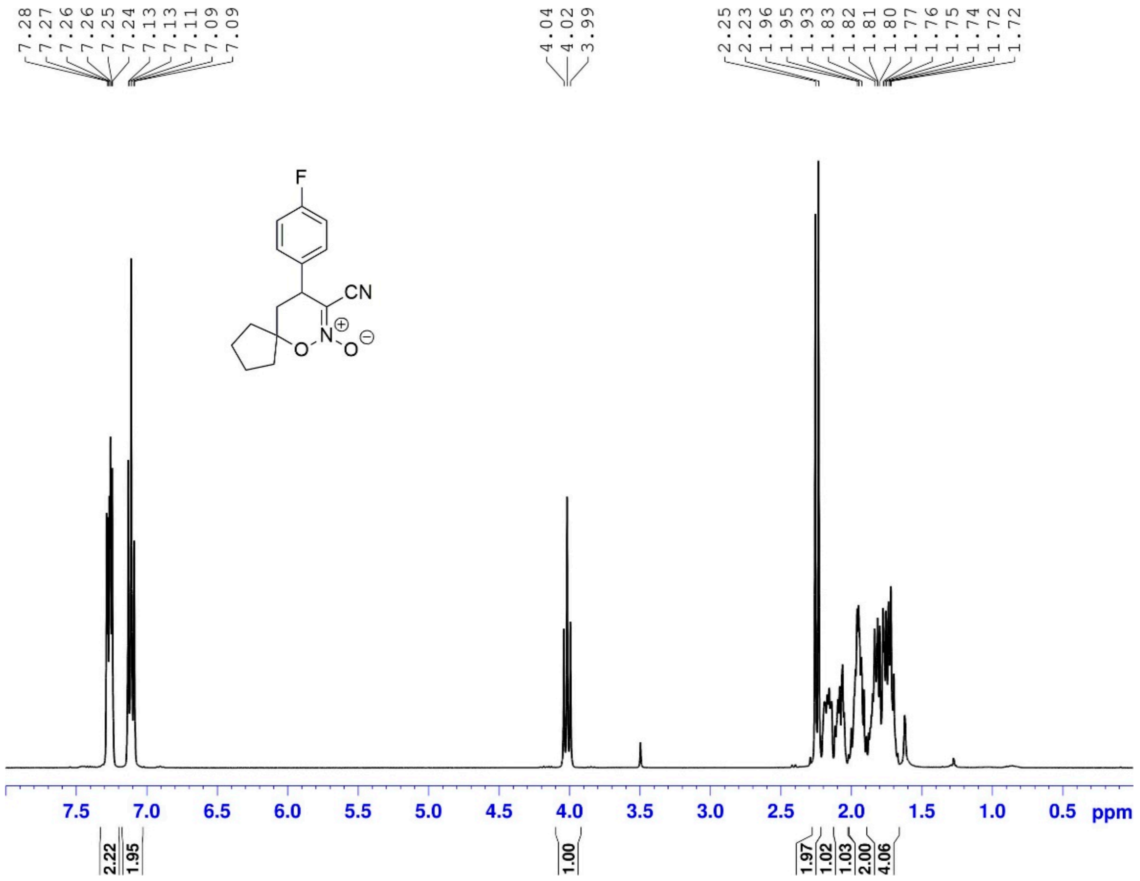
7.28
7.27
7.26
7.25
7.24
7.13
7.13
7.11
7.09
7.09
4.04
4.02
3.99
2.25
2.23
1.96
1.95
1.93
1.83
1.82
1.81
1.80
1.77
1.76
1.75
1.74
1.72
1.72
F
CN
N
O
O
7.5
7.0
6.5
6.0
5.5
5.0
4.5
4.0
3.5
3.0
2.5
2.0
1.5
1.0
0.5
ppm
2.22
1.95
1.00
1.97
1.02
1.03
2.00
4.06

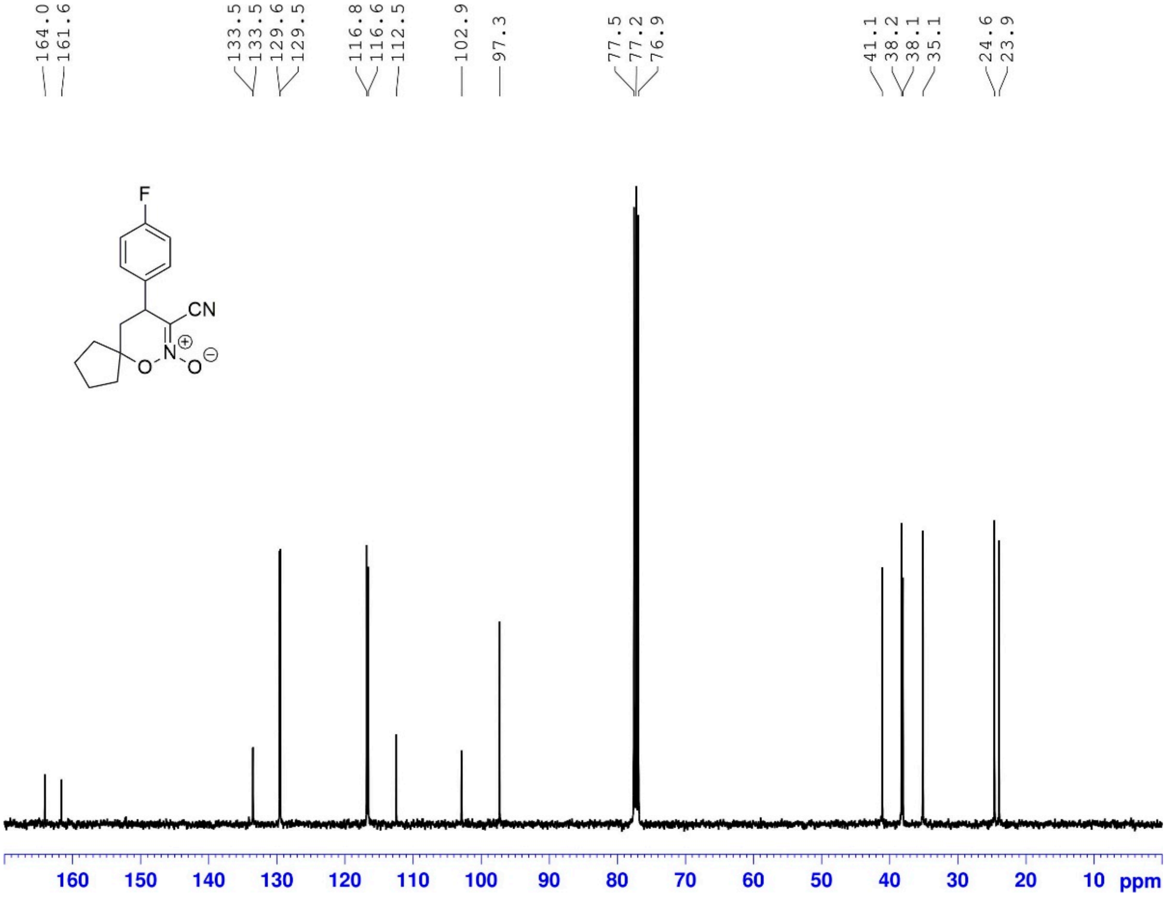
164.0
161.6
133.5
133.5
129.6
129.5
116.8
116.6
112.5
102.9
97.3
77.5
77.2
76.9
41.1
38.2
38.1
35.1
24.6
23.9
F
CN
N
O
O
160
150
140
130
120
110
100
90
80
70
60
50
40
30
20
10
ppm

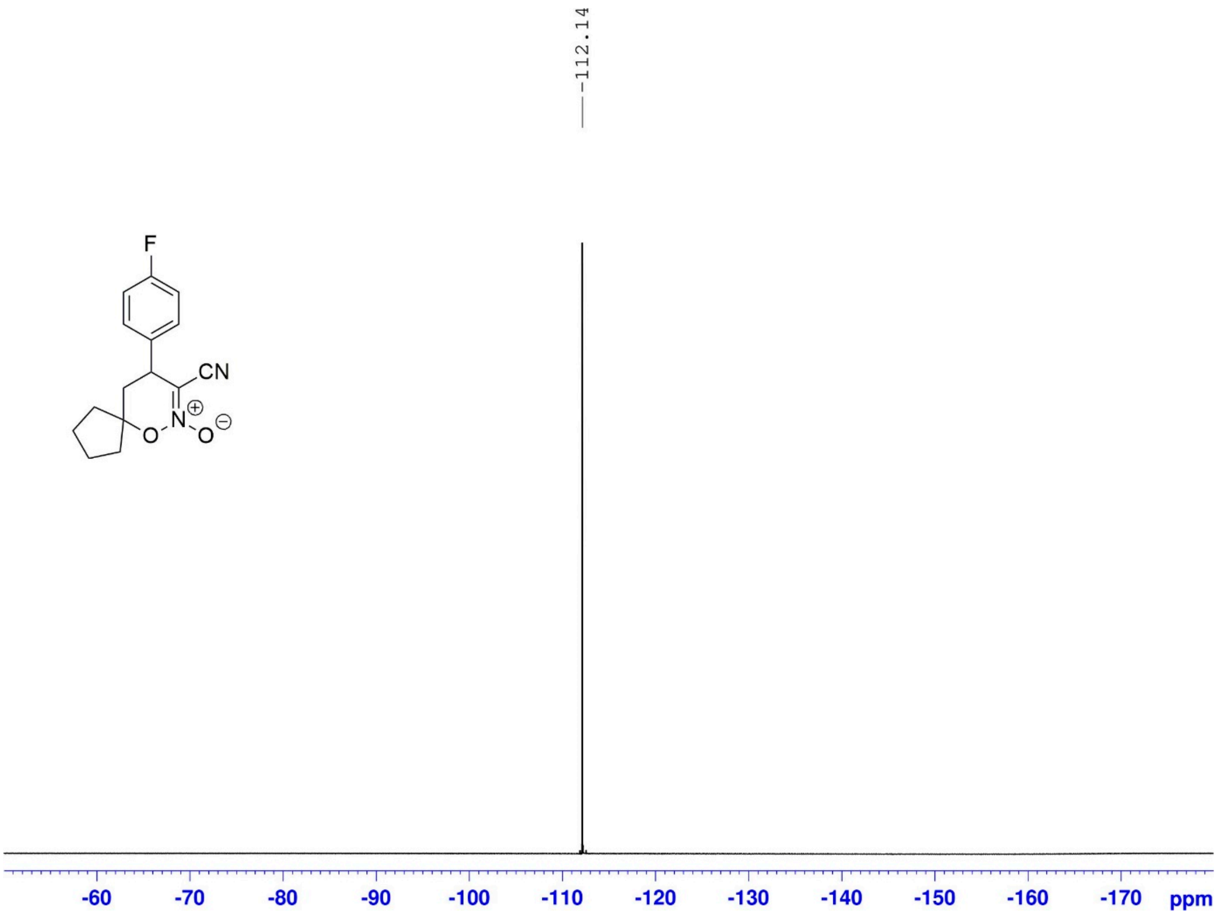
-112.14
F
CN
O
N
O
-60
-70
-80
-90
-100
-110
-120
-130
-140
-150
-160
-170
ppm

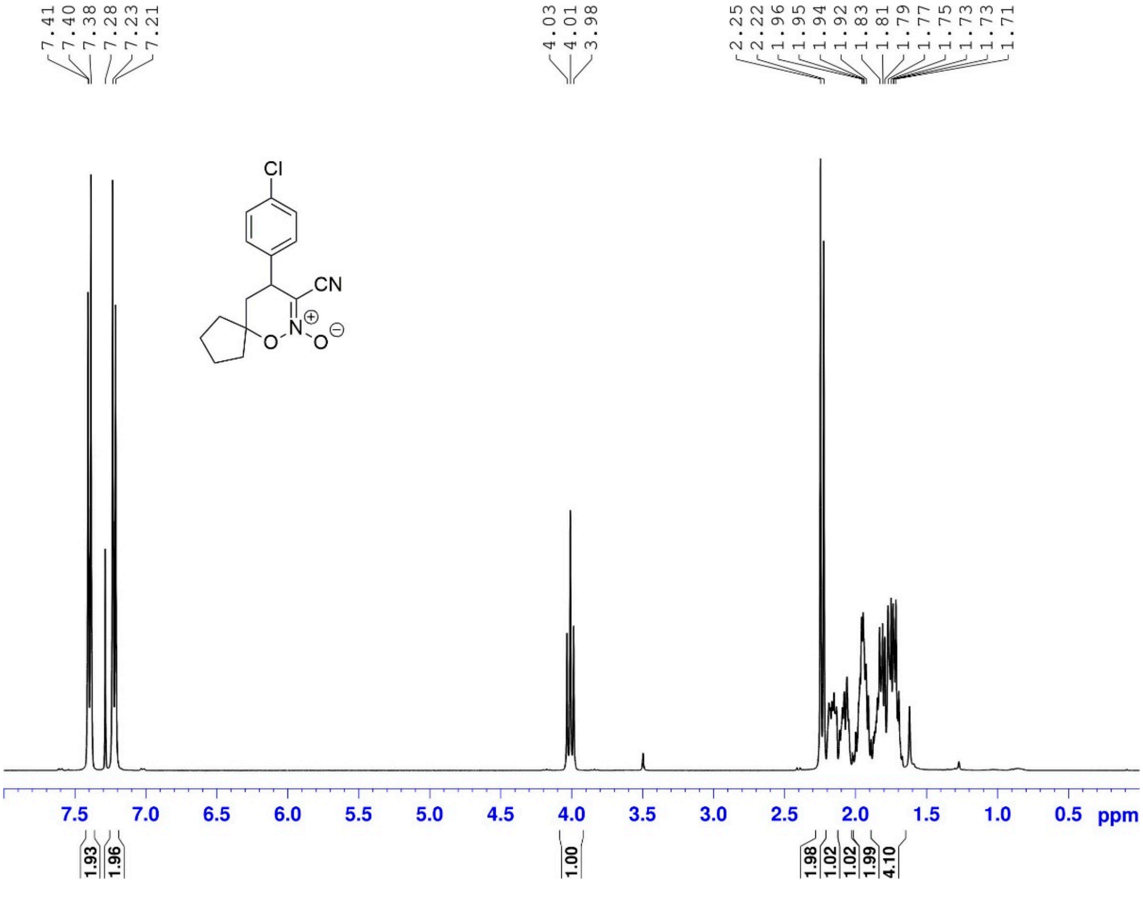
7.41
7.40
7.38
7.28
7.23
7.21
4.03
4.01
3.98
2.25
2.22
1.96
1.95
1.94
1.92
1.83
1.81
1.79
1.77
1.75
1.73
1.73
1.71
Cl
CN
O
N
O
7.5
7.0
6.5
6.0
5.5
5.0
4.5
4.0
3.5
3.0
2.5
2.0
1.5
1.0
0.5
ppm
1.93
1.96
1.00
1.98
1.02
1.02
1.99
4.10

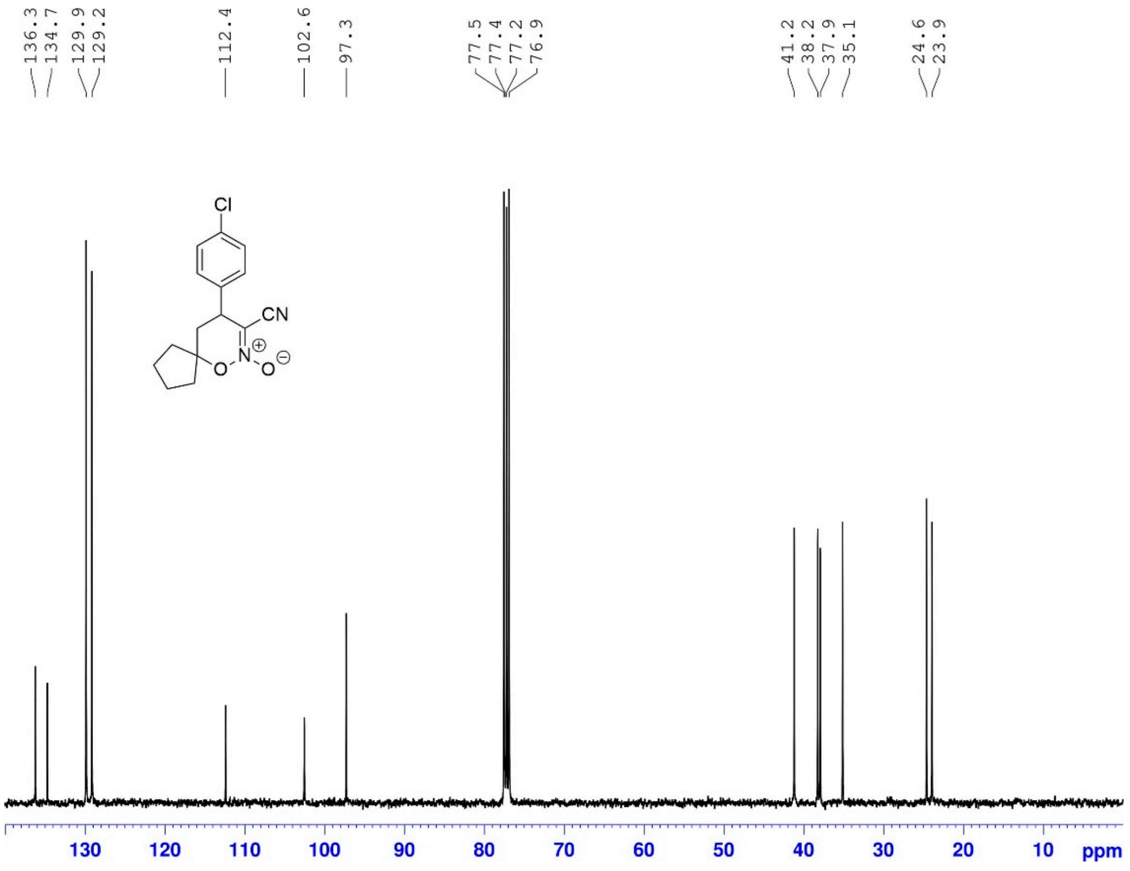
136.3
134.7
129.9
129.2
112.4
102.6
97.3
77.5
77.4
77.2
76.9
41.2
38.2
37.9
35.1
24.6
23.9
Cl
CN
N
O
O
130 120 110 100 90 80 70 60 50 40 30 20 10 ppm

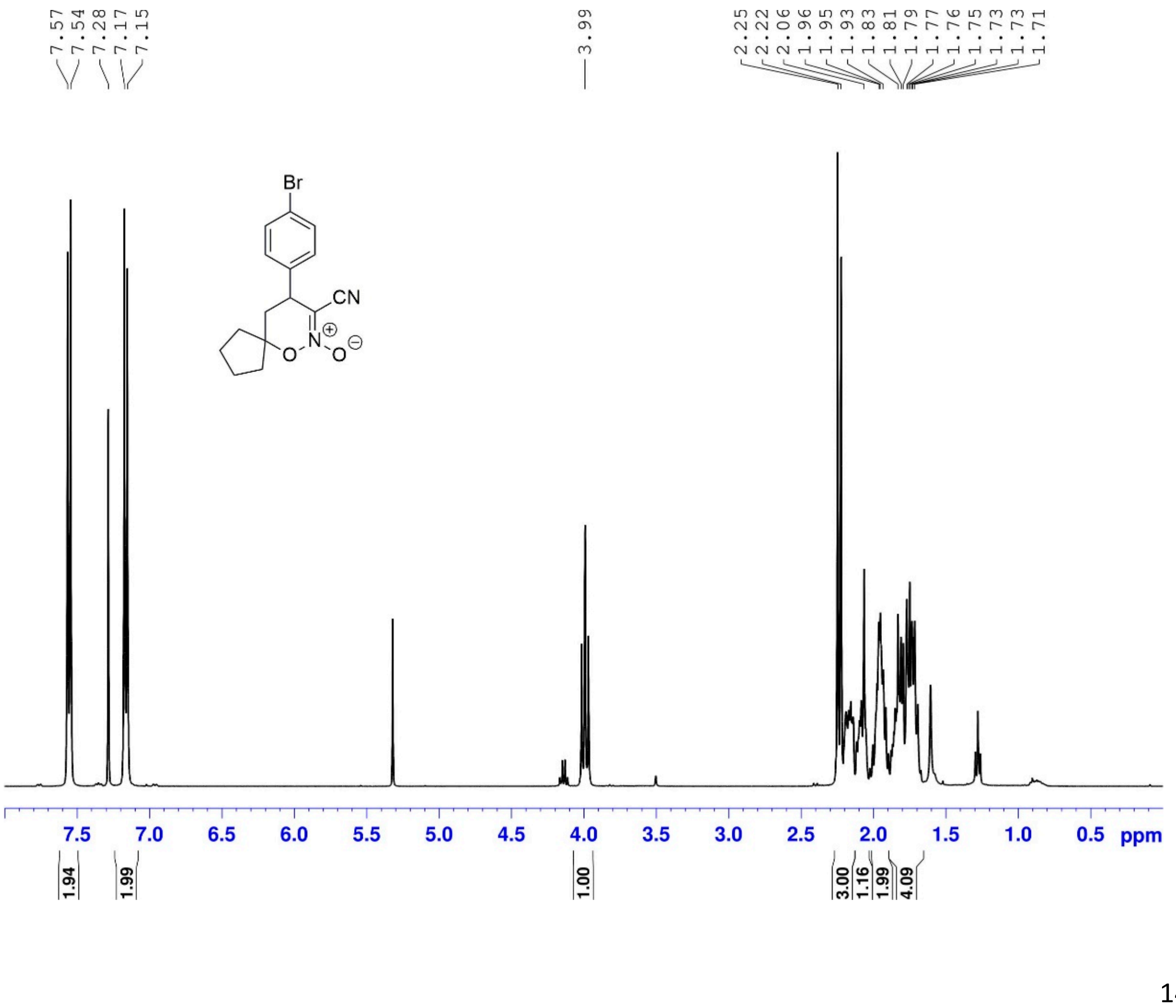
7.57
7.54
7.28
7.17
7.15
3.99
2.25
2.22
2.06
1.96
1.95
1.93
1.83
1.81
1.79
1.77
1.76
1.75
1.73
1.71
Br
CN
N
O
O
7.5 7.0 6.5 6.0 5.5 5.0 4.5 4.0 3.5 3.0 2.5 2.0 1.5 1.0 0.5 ppm
1.94
1.99
1.00
3.00
1.16
1.99
4.09

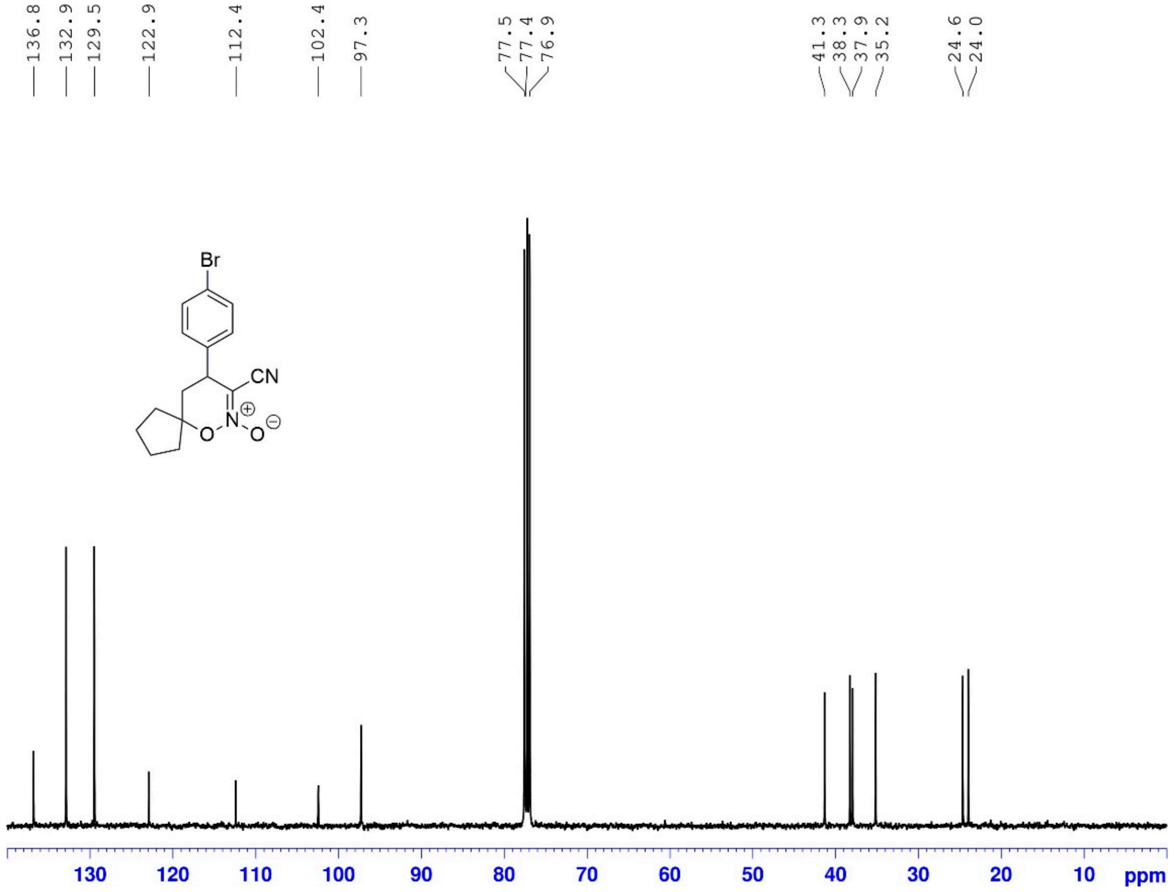
136.8
132.9
129.5
122.9
112.4
102.4
97.3
77.5
77.4
76.9
41.3
38.3
37.9
35.2
24.6
24.0
Br
CN
130 120 110 100 90 80 70 60 50 40 30 20 10 ppm

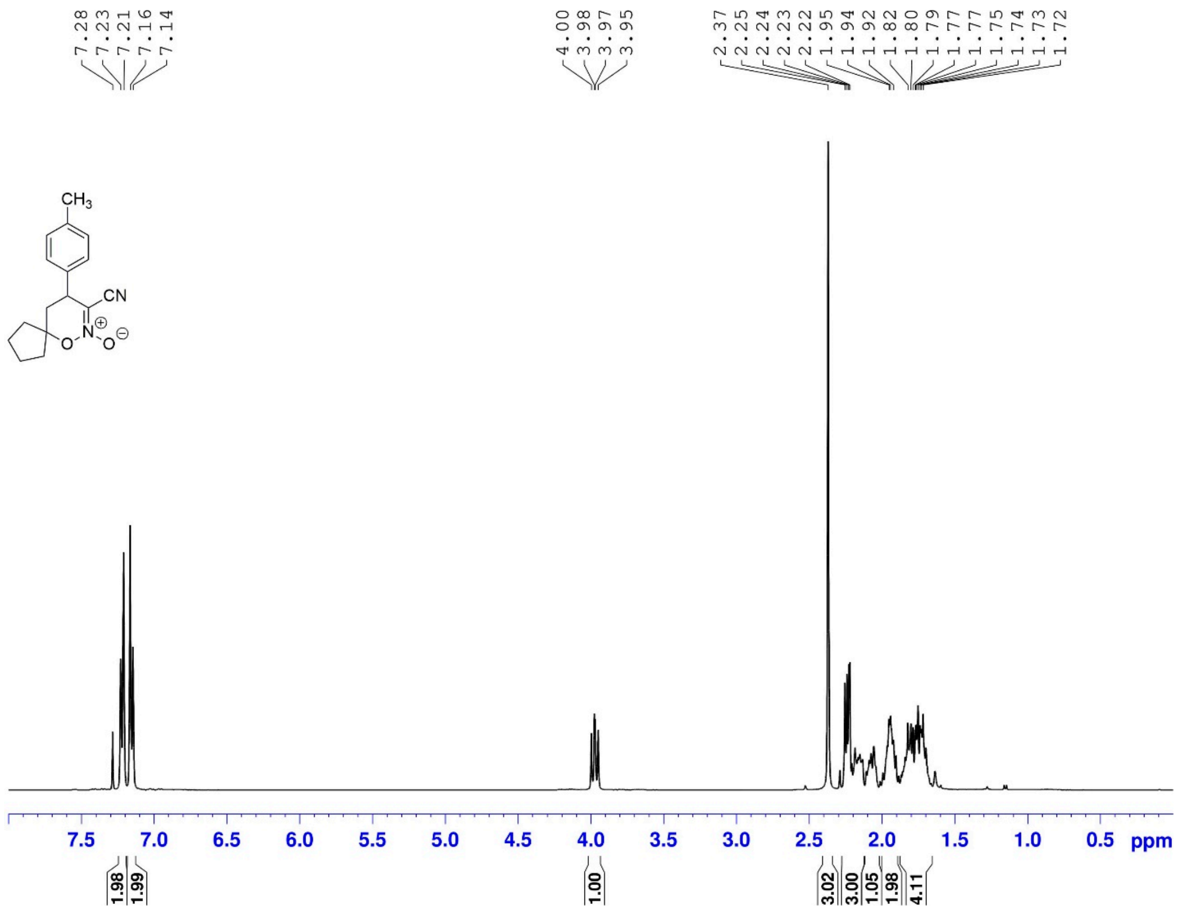
7.28
7.23
7.21
7.16
7.14
4.00
3.98
3.97
3.95
2.37
2.25
2.24
2.23
2.22
1.95
1.94
1.92
1.82
1.80
1.79
1.77
1.77
1.75
1.74
1.73
1.72
CH3
CN
7.5 7.0 6.5 6.0 5.5 5.0 4.5 4.0 3.5 3.0 2.5 2.0 1.5 1.0 0.5 ppm
1.98
1.99
1.00
3.02
3.00
1.05
1.98
4.11

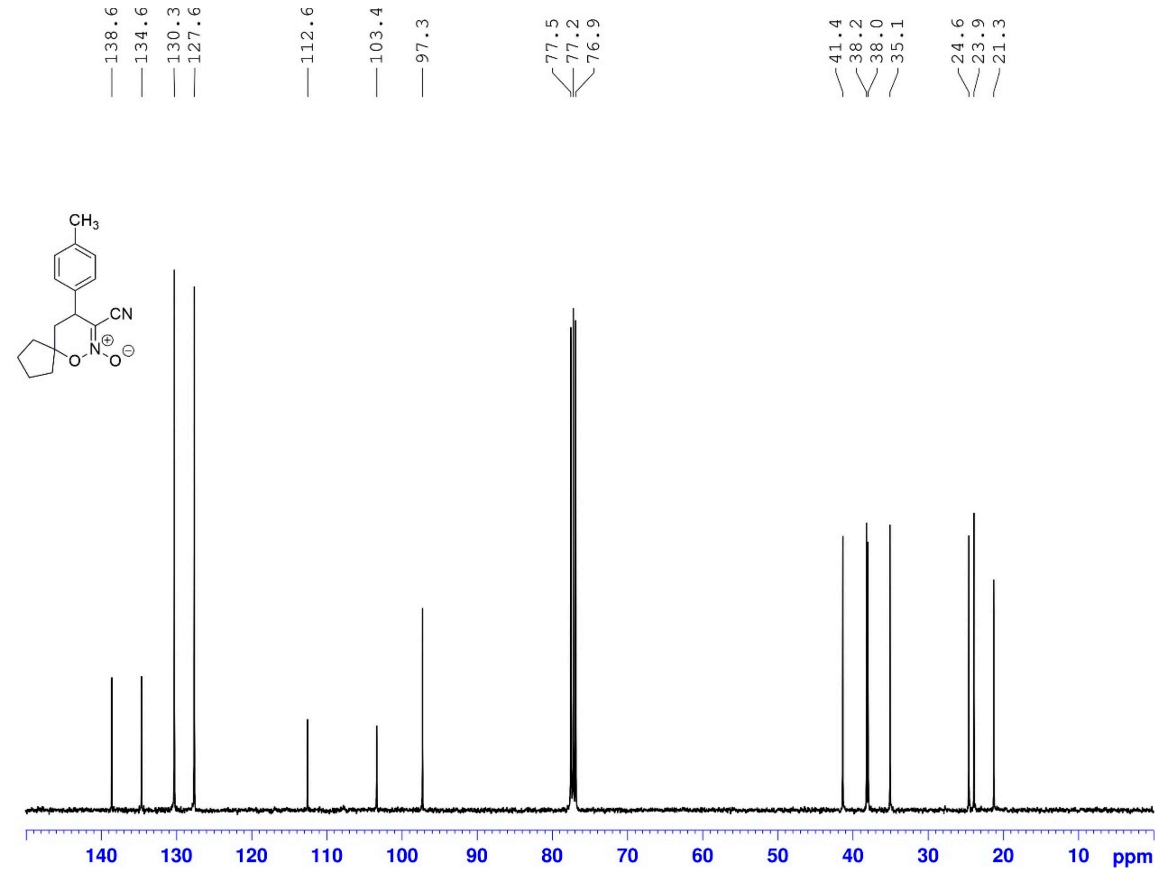
138.6
134.6
130.3
127.6
112.6
103.4
97.3
77.5
77.2
76.9
41.4
38.2
38.0
35.1
24.6
23.9
21.3
CH3
CN
O
N
140 130 120 110 100 90 80 70 60 50 40 30 20 10 ppm

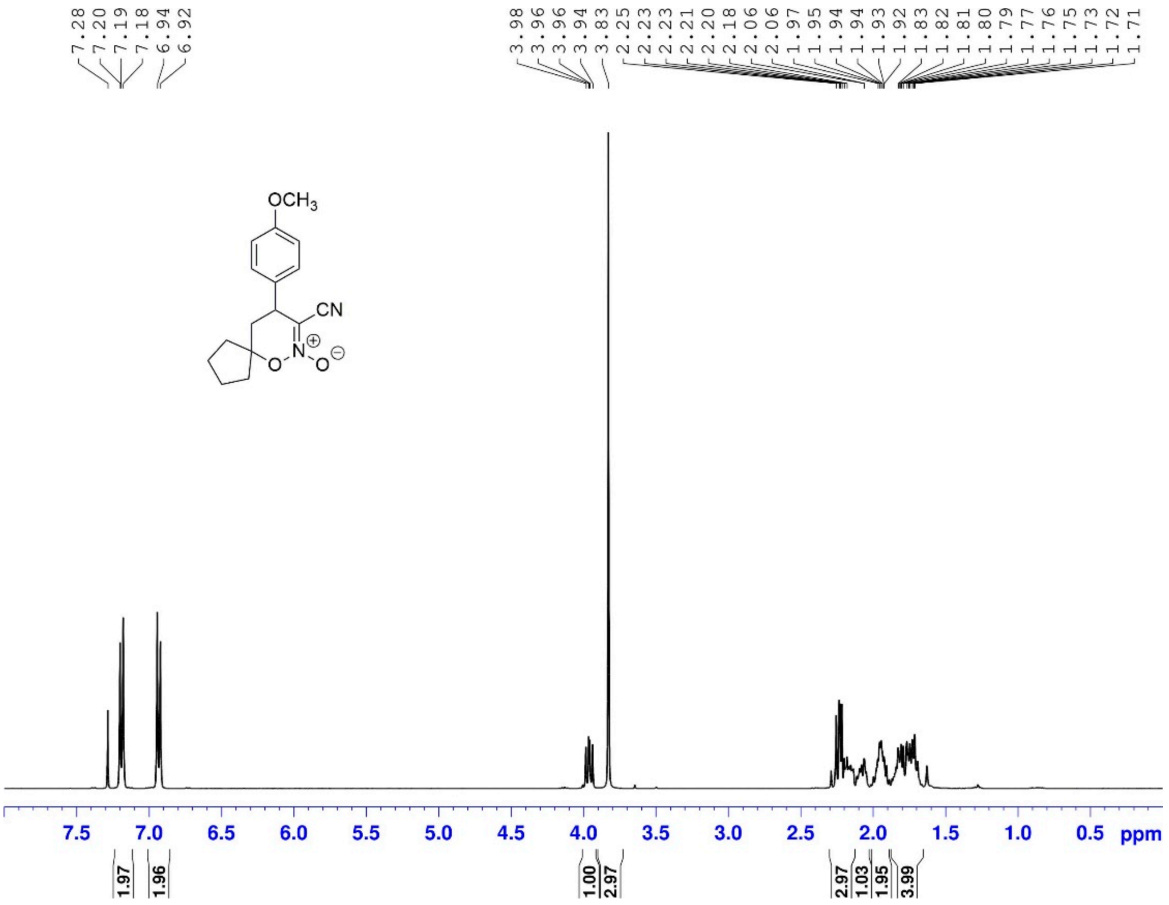
7.28
7.20
7.19
7.18
6.94
6.92
3.98
3.96
3.96
3.94
3.83
2.25
2.23
2.23
2.21
2.20
2.18
2.06
2.06
1.97
1.95
1.94
1.94
1.93
1.92
1.83
1.82
1.81
1.80
1.79
1.77
1.76
1.75
1.73
1.72
1.71
OCH3
CN
O
N
7.5 7.0 6.5 6.0 5.5 5.0 4.5 4.0 3.5 3.0 2.5 2.0 1.5 1.0 0.5 ppm
1.97
1.96
1.00
2.97
2.97
1.03
1.95
3.99

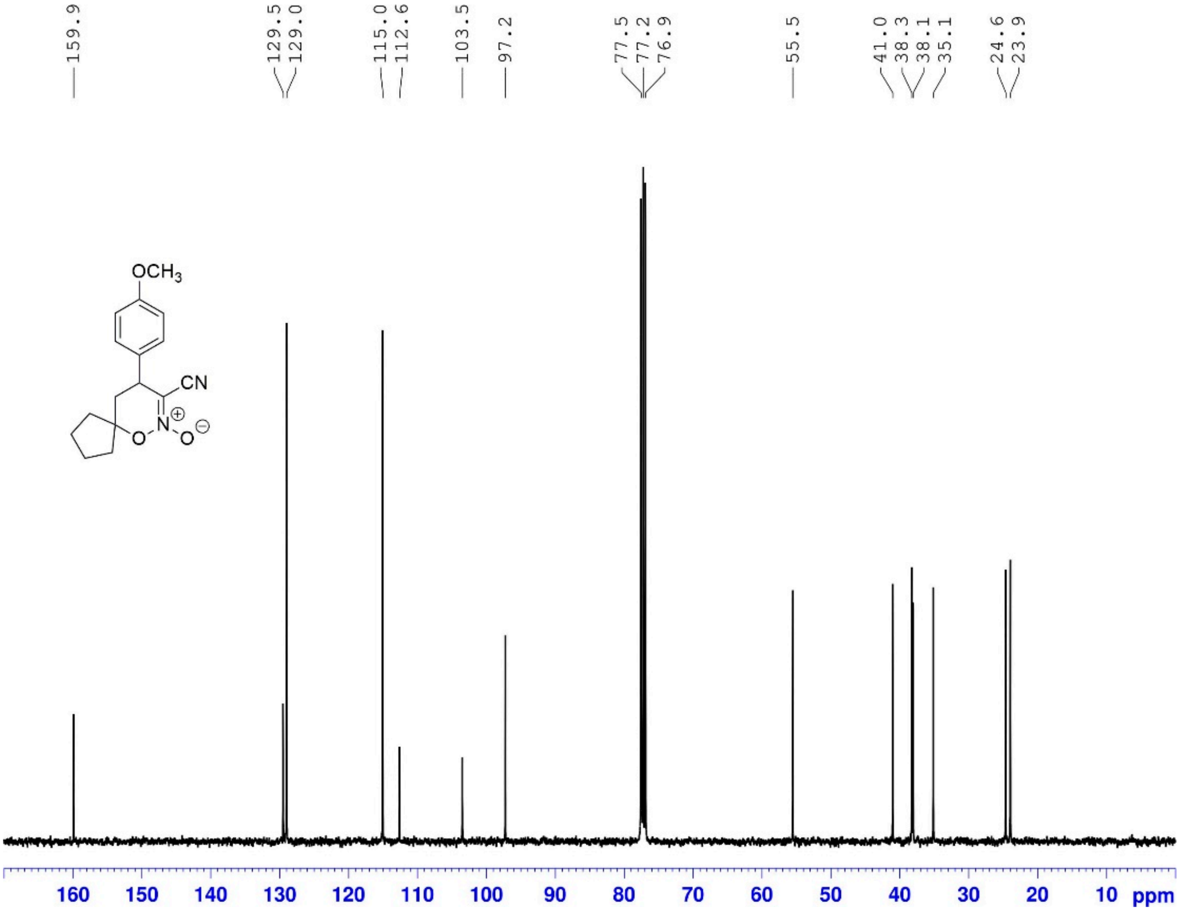
159.9
129.5
129.0
115.0
112.6
103.5
97.2
77.5
77.2
76.9
55.5
41.0
38.3
38.1
35.1
24.6
23.9
OCH3
CN
N
O
O
ppm

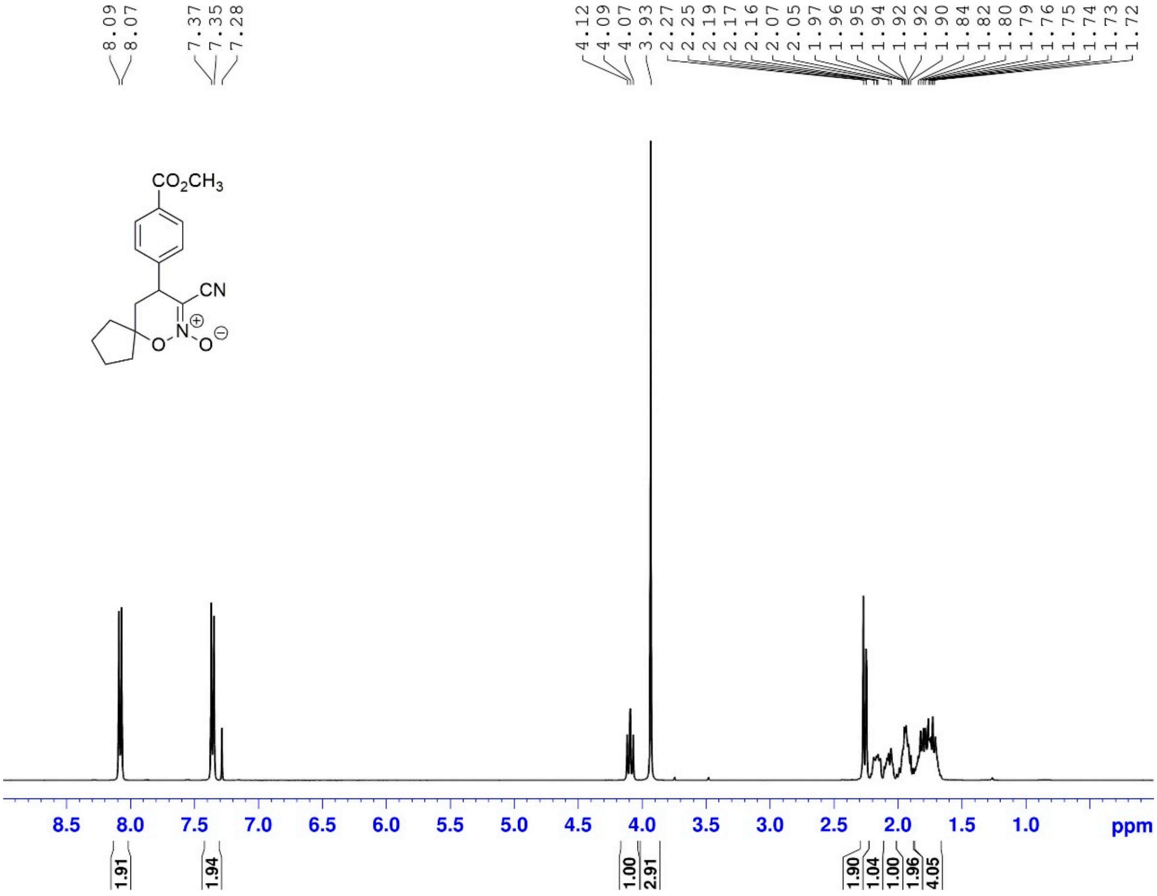
8.09
8.07
7.37
7.35
7.28
4.12
4.09
4.07
3.93
2.27
2.25
2.19
2.17
2.16
2.07
2.05
1.97
1.96
1.95
1.94
1.92
1.92
1.90
1.84
1.82
1.80
1.79
1.76
1.75
1.74
1.73
1.72
CO2CH3
CN
N
O
O
ppm
1.91
1.94
1.00
2.91
1.90
1.04
1.00
1.96
4.05

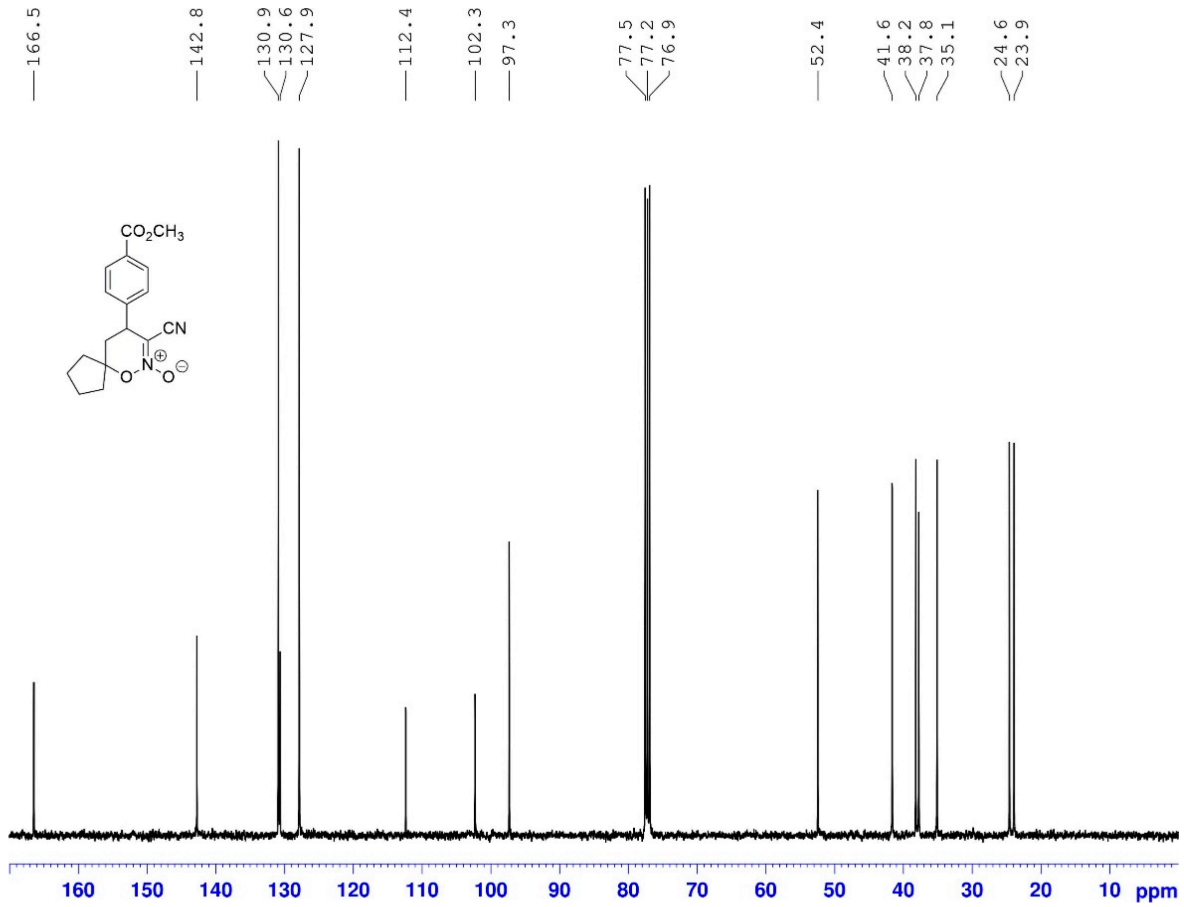

Table S1. The *m/z* values and a molecular formula of the major fragment ions in (-) APCI mass spectra of **3a-3g**.

	**[M − H⁺]⁻**	**[M-NO-H⁺]⁻**	**[M-C_5H_8O-H⁺]⁻**	**[M-$C_5H_7NO_2$-H⁺]⁻**
**3a**	285.1237 ($C_{16}H_{17}N_2O_3$)	256.1332 ($C_{16}H_{18}NO_2$)	201.0664 ($C_{11}H_9N_2O_2$)	172.0761 ($C_{10}H_{10}NO$)
**3b**	269.1291 ($C_{16}H_{17}N_2O_2$)	240.1391 ($C_{16}H_{18}N_2O_2$)	185.0717 ($C_{11}H_9N_2O$)	156.0820 ($C_{11}H_{10}N$)
**3c**	255.1138 ($C_{15}H_{15}N_2O_2$)	226.1238 ($C_{15}H_{16}NO$)	171.0565 ($C_{10}H_7N_2O$)	142.0664 ($C_{10}H_8N$)
**3d**	273.1044 ($C_{15}H_{14}N_2O_2F$)	244.1142 ($C_{15}H_{15}NOF$)	189.0470 ($C_{10}H_6N_2OF$)	160.0568 ($C_{10}H_7NF$)
**3e**	289.0746 ($C_{15}H_{14}N_2O_2Cl$)	260.0847 ($C_{15}H_{15}NOCl$)	205.0168 ($C_{10}H_6N_2OCl$)	176.0279 ($C_{10}H_7NCl$)
**3f**	333.0233 ($C_{15}H_{14}N_2O_2Br$)	304.0320 ($C_{15}H_{15}NOBr$)	248.9652 ($C_{10}H_6N_2OBr$)	219.9717 ($C_{10}H_7NBr$)
**3g**	313.1187 ($C_{17}H_{17}N_2O_4$)	284.1285 ($C_{17}H_{18}NO_3$)	229.0612 ($C_{12}H_9N_2O_3$)	200.0714 ($C_{12}H_{10}NO_2$)

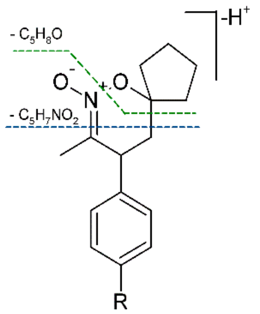

Table S2. Crystal structure, data collection and refinement parameters for 8-cyano-9-phenyl-6-oxa-7-aza-spiro-[4.5]dec-7-ene 7-oxide **3c**.

Chemical formula	$C_{15}H_{16}N_2O_2$
**Crystal data**	
Formula weight	256.30
Crystal system	Monoclinic
Space group	$P2_1/c$
Temperature (K)	100.00(10)
a [Å]	7.67046(4)
b [Å]	20.22880(13)
c [Å]	8.54662(4)
β [°]	94.9469(4)
V [Å^3]	1321.190(12)
Z	4
Z′	1
d_{calc} [g/cm^3]	1.289
Crystal dimensions [mm]	0.7 × 0.6 × 0.4
Radiation type	CuKα
μ [mm^{-1}]	0.700

**Data collection**	
Reflections measured	47881
Range/indices (h, k, l)	-9, 9; -25, 23; -10, 10
θ (max, min) [°]	75.8, 4.4
Total no. of unique data	2700
No. of observed data, I > 2σ(I)	2664
R_{int}	0.038

**Refinement**	
R [$F^2 > 2\sigma(F^2)$]	0.034
$wR(F^2)$	0.084
S	1.06
No. of reflections	2700
No. of parameters	173
No. of restraints	0
H-atom treatment	H atoms treated by constrained refinement
Δρ (min, max), e/Å^3	-0.19, 0.27

Table S3. Selected bond lengths and valence angles in 8-cyano-9-phenyl-6-oxa-7-aza-spiro-[4.5]dec-7-ene 7-oxide **3c** [Å, °].

**Length**		**Angle**	
O1—N2	1.3953 (11)	N2—O1—C6	115.02 (7)
O1—C6	1.4872 (11)	O11—N2—O1	113.06 (8)
N2—O11	1.2439 (11)	O11—N2—C3	126.63 (9)
C3—N2	1.3186 (13)	C3—N2—O1	120.28 (8)
C3—C4	1.5100 (13)	N2—C3—C12	114.42 (9)
C3—C12	1.4310 (14)	N2—C3—C4	125.46 (9)
C4—C5	1.5331 (14)	C12—C3—C4	120.11 (9)
C4—C14	1.5214 (14)	C15—C14—C4	120.70 (9)
C5—C6	1.5149 (14)	C19—C14—C4	120.13 (9)
C6—C7	1.5232 (14)	C19—C14—C15	119.16 (10)
C6—C10	1.5261 (14)	N13—C12—C3	176.34 (11)
C7—C8	1.5445 (16)	C3—C4—C14	110.23 (8)
C8—C9	1.5425 (17)	C3—C4—C5	110.03 (8)
C9—C10	1.5341 (15)	C14—C4—C5	112.06 (8)
C12—N13	1.1489 (14)	C16—C15—C14	120.55 (10)
C14—C15	1.3950 (15)	C6—C5—C4	110.76 (8)
C14—C19	1.3902 (15)	C17—C16—C15	119.93 (10)
C15—C16	1.3889 (15)	O1—C6—C5	107.13 (8)
C16—C17	1.3885 (16)	O1—C6—C10	109.06 (8)
C17—C18	1.3830 (16)	O1—C6—C7	102.62 (8)
C19—C18	1.3948 (15)	C5—C6—C10	117.95 (9)
		C5—C6—C7	115.10 (9)
		C7—C6—C10	103.85 (8)
		C18—C17—C16	119.87 (10)
		C6—C10—C9	103.33 (9)
		C14—C19—C18	120.13 (10)
		C6—C7—C8	106.33 (9)
		C17—C18—C19	120.31 (10)
		C9—C8—C7	105.86 (9)
		C10—C9—C8	104.72 (9)
